# Molecular Characterization and Expression Profiling of *Brachypodium distachyon* L. Cystatin Genes Reveal High Evolutionary Conservation and Functional Divergence in Response to Abiotic Stress

**DOI:** 10.3389/fpls.2017.00743

**Published:** 2017-05-09

**Authors:** Saminathan Subburaj, Dong Zhu, Xiaohui Li, Yingkao Hu, Yueming Yan

**Affiliations:** College of Life Science, Capital Normal UniversityBeijing, China

**Keywords:** *Brachypodium distachyon* L., *BdC* genes, phylogenetic relationships, expression profiling, abiotic stress, qRT-PCR

## Abstract

Cystatin is a class of proteins mainly involved in cysteine protease inhibition and plant growth and development, as well as tolerance under various abiotic stresses. In this study, we performed the first comprehensive analysis of the molecular characterization and expression profiling in response to various abiotic stresses of the cystatin gene family in *Brachypodium distachyon*, a novel model plant for *Triticum* species with huge genomes. Comprehensive searches of the *Brachypodium* genome database identified 25 *B. distachyon cystatin* (*BdC*) genes that are distributed unevenly on chromosomes; of these, nine and two were involved in tandem and segmental duplication events, respectively. All *BdC* genes had similar exon/intron structural organization, with three conserved motifs similar to those from other plant species, indicating their high evolutionary conservation. Expression profiling of 10 typical *BdC* genes revealed ubiquitous expression in different organs at varying expression levels. *BdC* gene expression in seedling leaves was particularly highly induced by various abiotic stresses, including the plant hormone abscisic acid and various environmental cues (cold, H_2_O_2_, CdCl_2_, salt, and drought). Interestingly, most *BdC* genes were significantly upregulated under multiple abiotic stresses, including *BdC15* under all stresses, *BdC7-2* and *BdC10* under five stresses, and *BdC7-1, BdC2-1, BdC14*, and *BdC12* under four stresses. The putative metabolic pathways of cytastin genes in response to various abiotic stresses mainly involve the aberrant protein degradation pathway and reactive oxygen species (ROS)-triggered programmed cell death signaling pathways. These observations provide a better understanding of the structural and functional characteristics of the plant cystatin gene family.

## Introduction

Cystatins, which constitute a multigene family, form a class of proteins that inhibits cysteins proteases (Turk and Bode, 1991). Cystatins are sub-divided into stefins without disulfide bonds (family 1), cystatins with two disulfide bonds (family 2), and kininogens with nine disulfide bonds (family 3) based on primary sequence homology (Abrahamson et al., 2003). Most cystatins inhibit the activities of cathepsin L-like proteases, a cysteine protease in the peptidase C1A family (Martinez et al., 2009). Cystatins are widely distributed in both animal and plant systems (Margis et al., 1998; Kotsyfakis et al., 2006). Plant cystatins, referred to as phytocystatins (phy-cys), are small in size, about 12–16 kDa, and have the LARFAV consensus sequence motif in the region corresponding to a predicted N-terminal α-helix (Misaka et al., 1996). Additionally, phy-cys are believed to contain either N or C-terminal extensions that apparently raise their molecular weights up to 25 kDa (Misaka et al., 1996; Martinez et al., 2005). It has been suggested that phy-cys with short N-terminal and longer C-terminal extensions inhibit the activities of cysteine proteases in the peptidase C13 family (Martinez et al., 2007). There are three important signature motifs necessary for the protease inhibition reactions present in all cystatins: a QxVxG reactive site, one or two glycine (G) residues in the N-terminal part of the protein, and a tryptophan residue (W) located downstream of the reactive site (Margis et al., 1998).

Phy-cys have been reported in a wide range of plant species, including tomato (Wu and Haard, 2000), potato (Bouchard et al., 2003), sesame (Shyu et al., 2004), amaranth (Valdes-Rodriguez et al., 2007), alfalfa (Rivard et al., 2007), *Arabidopsis* (Zhang et al., 2008), sea rocket (Megdiche et al., 2009), and rice (Wang et al., 2015), etc. The functional roles of these phy-cys are well described and mostly involve plant growth and development, including fruit development (Neuteboom et al., 2009), seed development and germination (Hong et al., 2007; Hwang et al., 2010), and defense against pathogens and insects (Belenghi et al., 2003; Konrad et al., 2008). Phy-cys are ubiquitously expressed in a wide range of tissues and organs (Abraham et al., 2006; Valdes-Rodriguez et al., 2007). Additionally, phy-cys are also implicated in responses to adverse environmental stress, as observed by their transcript accumulation under different abiotic stress conditions, such as drought, salt, heat, oxidant stress, and cold (Valdes-Rodriguez et al., 2007; Zhang et al., 2008; Huang et al., 2012; Sun et al., 2014; Tan et al., 2014). Recent studies have found that over-expression of phy-cys enhances tolerance against abiotic stresses, such as alkali (Sun et al., 2014), drought (Tan et al., 2015), and heat (Je et al., 2014). Additionally, cystatins are involved in programmed cell death (PCD) through their inhibitory action against cysteine protease, which is mostly activated by abiotic stresses (Solomon et al., 1999; Belenghi et al., 2003). Ectopically expressed phy-cys in transgenic plants suggests that these genes could be useful for improving seed traits and delayed sprouting in agronomically important crops (Quain et al., 2014; Munger et al., 2015).

*Brachypodium distachyon* L., a temperate wild annual grass in the Pooideae subfamily has emerged as a novel model plant in the study of temperate cereals, such as wheat and related species (Draper et al., 2001). Although cystatin proteins have been investigated in some plant species, information on this gene family in *B. distachyon* is limited. Genome-wide identification and characterization of cystatin genes in *B. distachyon* are necessary to determine their functional roles in plant developmental processes and in defense against abiotic stress, which will help to improve cereal crop resistance to various stresses. In the present study, we provide the first molecular characterization and expression profiling of the *B. distachyon* cystatin genes in various tissues and examine their reactions under different abiotic stresses. Our findings provide novel insights into the structure, evolution, and function of the plant cystatin gene family.

## Materials and methods

### Retrieval and identification of cystatin gene sequences

To obtain the *B. distachyon* cystatin genes, previously published orthologous cystatin gene sequences from *Hordeum vulgare* (Martinez et al., 2009), *Oryza sativa* (Wang et al., 2015), *Triticum aestivum* (Kuroda et al., 2001), *Zea mays* (Massonneau et al., 2005), and *Sorghum bicolor* (Li et al., 1996) are listed in Table S1, which were used to BLAST against the *Brachypodium distachyon* genome database, Phytozome v9.0 (http://www.phytozome.net) by the BLAST program. Sequences were selected as candidate genes if they were described as cysteine protease inhibitor activity along with their *E*-value <10e–10. For each query sequence, information of the location on chromosomes, genomic sequences, full coding sequences (CDS), and protein sequences were collected from Phytozome. Unique cystatin genes were obtained by manually excluding the redundant sequences. Eventually, the identified candidate genes were named as *Brachypodium distachyon* cystatin (*BdC*). The putative cystatin protein sequences of *B. distachyon* are further analyzed with the InterPro program using the PFAM database (http://pfam.sanger.ac.uk; Bateman et al., 2002) and their cystatin domains deduced. Following the PFAM search, *BdC* genes without typical domain (Aspartic acid proteinase inhibitor) and reactive site motif (QxVxG; Margis et al., 1998) of cystatin protein were deleted from further analysis.

### Chromosomal locations, exons/introns organization, conserved motif analyses, and characteristics of cystatin genes

The gene locations were based on the Phytozome v9.1 database and mapped by MapInspect software. Identification and cataloging of *B. distachyon* cystatin genes in terms of intra-genome or cross-genome syntenic relationships were conducted using the Plant Genome Duplication Database (PGDD) (http://chibba.agtec.uga.edu/duplication/). The genomic organization such as exons and introns were analyzed by Gene Structure Display Server (GSDS; Guo et al., 2007). Analysis of conserved motifs was performed by MEME (Multiple Em for Motif Elicitation) software version 3.5.4 (Bailey et al., 2006) (http://meme-suite.org) using minimum and maximum motif width of 8 and 15 residues respectively, and a maximum number of 15 motifs, keeping the rest of the parameters at default. The protein sequence characteristics such as pI/Mw and signal peptide was predicted, respectively by using Compute pI/Mw tool (Gasteiger et al., 2005) and Signal P4.1 (Petersen et al., 2011). The subcellular distribution of the proteins was predicted by using TargetP 1.1 (www.cbs.dtu.dk/services/TargetP/) server.

### Multiple sequence alignment, hierarchical cluster analysis, tertiary structure prediction, and promoter analysis of cystatin genes

Analysis of DNA and comparisons of deduced protein sequences alignments were carried out by BioEdit software (Hall, 1999). Hierarchical clustering of cystatins was performed by MultiAlin tool using alignment parameters of identity, gap penalty at 8 and 2, respectively for opening and extension (Corpet, 1988). The three-dimensional structures of the *B. distachyon* cystatins were modeled by Phyre2 Server (http://www.sbg.bio.ic.ac.uk/phyre2/html/) (Kelley and Sternberg, 2009). Promoter sequences of cystatins were examined using plantCARE database (http://bioinformatics.psb.ugent.be/webtools/plantcare/html/) (Lescot et al., 2002). A stretch of 2,000 bases upstream of the start site was considered for analysis.

### Phylogenetic analysis

A total of 71 cystatin sequences those from 7 plant species including *Brachypodium* in this study were used to construct a phylogenetic tree. The sequences and respective protein ID or transcript names are displayed in Table S1, and the corresponding nomenclatures were composed of two letters for genus and species, followed by *BdC* and an Arabic number. The cystatin amino acid sequences of the whole coding regions were aligned by ClustalW parameters using the Gonnet series as the protein weight matrix. Phylogenetic analysis of the sequences was done by MEGA (Molecular Evolutionary Genetic Analysis) software 5.10 (Tamura et al., 2011) using the neighbor-joining (NJ) method with complete deletion, JTT matrix-based method (Jones et al., 1992) and 1,000 bootstrap replicates with the bootstrap method.

### Plant growth, stress treatments and sample collection

*Brachypodium distachyon* 21 (Bd21) seeds were sterilized with 75% alcohol and 15% sodium hypochlorite, rinsed 4–5 times and placed on moistened filter paper in Petri dishes and germinated at 26°C for 1 week. Then the seedlings were transferred to plastic pots (ten seedlings per pot) filled with Hoagland solution in a growth room at 22°C and a 16 h day/8 h night photoperiod and supplemented with an average cool white fluorescent light photon flux of 180 μmol s^−1^ m^−2^. The nutrient solution in pots were routinely changed every 3 days. When the seedlings reached the two-leaf stage, various stresses such as cold, H_2_O_2_, CdCl_2_, drought, salinity, and ABA treatments were initiated according to the procedures described in previous reports (Lv et al., 2014; Zhu et al., 2015). For each treatment, three pots were used. Cold stress was provided to seedlings by placing them in a growth chamber with 4°C for 12 and 24 h. CdCl_2_ stress were investigated with 50 μM for 6 and 12 h. Drought treatment was carried out with 200 mM polyethylene glycol 6,000 for 12 and 24 h; salt treatment accomplished with 160 mM sodium chloride treatment for 12 and 24 h; 0.1 mM ABA treatment done for 6 h; and 20 mM H_2_O_2_ treatment was for 2, 4, and 6 h. For each experimental conditions either for stress treatment and control plants, triplicates (three biological replicates) were used. After the stress treatment, control and treated leaves were harvested for assays. Developing caryopses were sampled from 4 to 30 days post anthesis (DPA) at 2–5 day intervals (12 sampling times) according to a previous study (Chen et al., 2014). All samples were immediately frozen in liquid nitrogen and kept at −80°C prior to RNA isolation.

### mRNA isolation, cDNA synthesis and quantitative real-time polymerase chain reaction (qRT-PCR)

Total mRNA was extracted by using the Trizol method. Transcript levels of cystatins genes were analyzed at relative levels by using the SYBR Green-based qRT-PCR method (CFX96, Bio-Rad Thermal Cycler system C1000 series). A 20 μl of reaction volume containing cDNA template, 2 × SYBR premix Ex Taq, 0.5 μM oligonucleotide primer (Takara, SYBR Ex Taq, China) was used for qRT-PCR. The PCR reaction included one cycle at 95°C for 3 min, followed by 39 cycles of 95°C for 15 s, 60°C for 20 s and 72°C for 15 s, and a final cycle at 65°C for 5 s and 95°C for 2 s to check the specificity of the oligonucleotides annealing and dissociation kinetics. The *Ubiquitin* gene from *Brachypodium* was used as reference genes according to previous reports (Hong et al., 2008). Primer pairs for qRT-PCR analysis (Table S2) were designed by the Primer3Plus program (http://www.bioinformatics.nl). Gene-specific amplification of both target and reference genes were standardized by the presence of a single, dominant peak in the qRT-PCR dissociation curve analyses. The relative expression level of cystatin mRNA transcripts was calculated relative to *Ubiquitin* by using the comparative threshold cycle method (Pfaffl, 2001). To determine the number of cDNA copies of duplicated *cystatin* genes, absolute mRNA expression levels of five genes were measured by construction of standard curve with serial dilutions of known amount of linearised plasmid DNA carrying target (*Cystatin*) and reference (*Ubiquitin*) gene, in which transcript or cDNA copies of each target genes were estimated according to previous report (d'Aloisio et al., 2010; Subburaj et al., 2014). The amplification efficiencies (E) and R2-values (coefficient of determination) of both target and reference genes were generated using the slopes of the standard curves obtained by serial dilutions. The efficiency range of the qRT-PCR amplifications for all of the genes tested was between 90 and 110%. All qRT-PCRs were carried out for three technical and three biological replicates and were normalized according to previous reports (Pfaffl, 2001; Hong et al., 2008).

## Results

### *In silico* identification and genomic distribution of cystatin genes in *B. distachyon*

**Figure 1:**
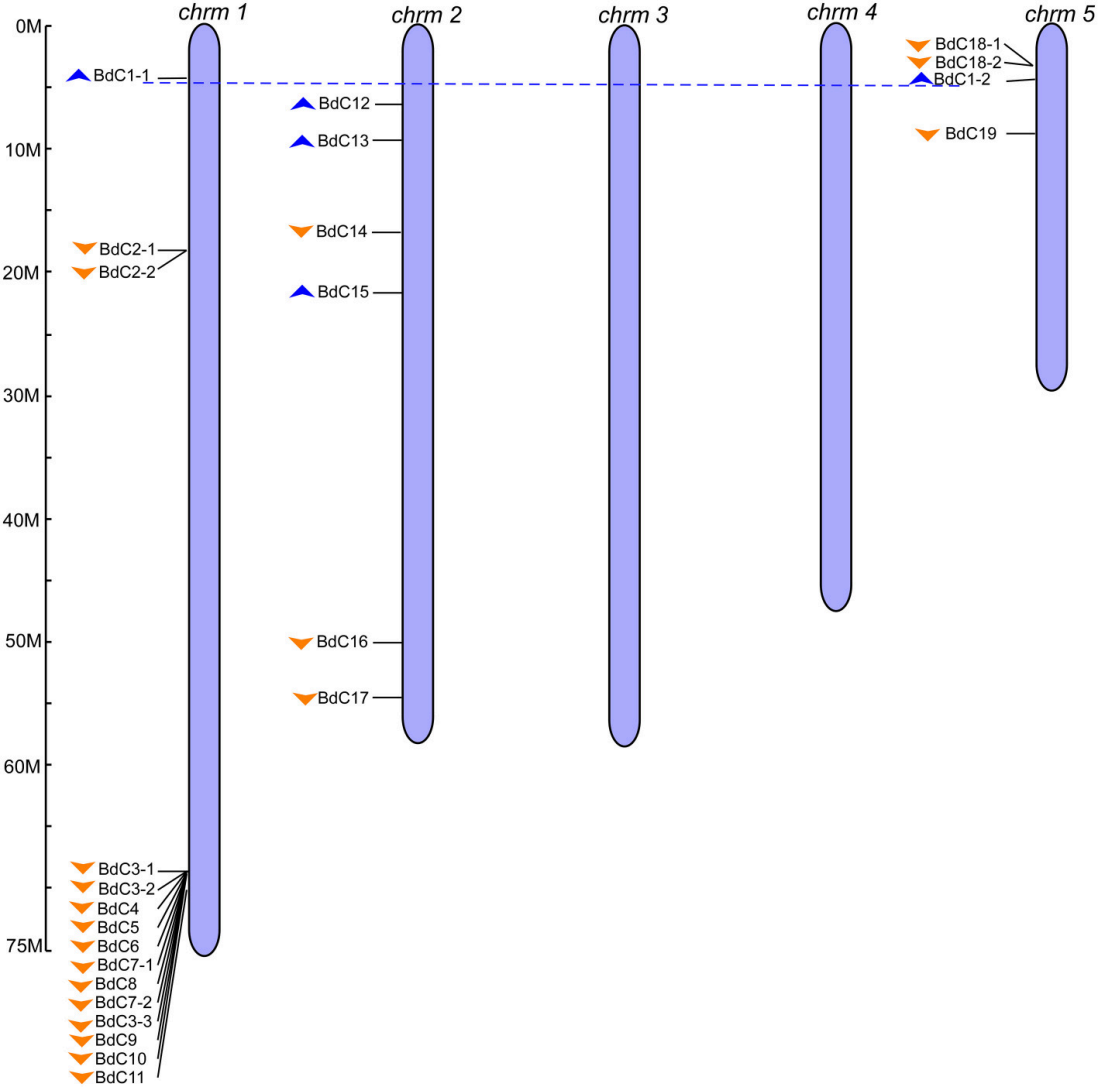
**Genomic distribution of *B. distachyon* cystatin (*BdC*) genes**. Chromosome numbers are indicated at the top of each bar and the scales show their each size (Mb). Blue and orange triangles indicate the upward and downward direction of transcription, respectively. Blue dotted lines connect the *BdC* genes present on duplicate chromosomal segments. The ruler represented in mega bases (M).

To obtain *B. distachyon* cystatin (*BdC*) genes, previously characterized cystatin sequences from wheat, rice, barley, and maize were used as queries to search the public *Brachypodium* genome database in Phytozome v9.0 (http://www.phytozome.org/). A total of 25 non-redundant *BdC* genes and their protein encoding sequences were identified (Table S3) and serially named *BdC1*–*BdC19* based on their location and chromosomal order (Table 1). Chromosomal distribution showed that *BdC* genes are dispersed over several chromosomes (Figure 1), and the number of *BdC* genes distributed per chromosome (Nos. 1 to 5) was 15, 6, 0, 0, and 4, respectively. The highest *BdC* density was found on chromosome 1, with lower densities on chromosomes 2 and 5, whereas chromosomes 3 and 4 had no cystatin genes.

**Table 1:** **Physiochemical, structural, and sequence properties of 25 members of *BdC* gene family identified in *Brachypodium distachyon* L**.

**Sequence ID**	**Gene name**	**Genomic (bp)**	**CDS Length (bp)**	**Residue length (aa)**	**Theoretical MW**	***pI***	**TargetP Predicted Location^a^**	**QxVxG Motif^b^**
Bradi1g02200	*BdC1-1*	426	426	141	14.93	9.20	S/2	QLVPV
Bradi1g22700	*BdC 2-1*	592	387	128	13.50	10.72	S/3	QVVAG
Bradi1g22710	*BdC 2-2*	372	372	123	13.35	4.74	S/1	QVVAG
Bradi1g70100	*BdC 3-1*	351	351	116	12.98	8.02	C/2	QELSE
Bradi1g70110	*BdC 3-2*	351	351	116	12.98	8.02	S/2	QDLST
Bradi1g70480	*BdC 4*	351	351	116	12.56	9.13	S/1	LVLVG
Bradi1g70490	*BdC 5*	345	345	114	12.50	5.63	S/1	QKLPN
Bradi1g70500	*BdC 6*	533	348	115	12.47	9.13	S/1	QVVAG
Bradi1g70510	*BdC 7-1*	339	339	112	12.12	7.90	S/1	QDVAG
Bradi1g70520	*BdC 8*	357	357	118	12.36	9.52	S/1	QIVAG
Bradi1g70530	*BdC 7-2*	348	348	115	12.60	9.13	S/1	QDVVG
Bradi1g70540	*BdC 3-3*	348	348	115	12.67	8.03	S/1	QQLLT
Bradi1g70550	*BdC 9*	402	402	133	14.10	5.73	S/1	QIGAD
Bradi1g70560	*BdC 10*	390	390	129	14.85	9.69	S/1	QAVES
Bradi1g73090	*BdC 11*	630	429	142	15.13	9.22	S/1	QPRVD
Bradi2g10140	*BdC 12*	1884	513	170	18.40	5.80	S/3	EVVED/ DPVVK
Bradi2g12750	*BdC 13*	369	369	122	13.13	4.96	S/2	QAVTE
Bradi2g21280	*BdC 14*	698	429	142	15.80	10.20	?/4	QLVASG
Bradi2g25507	*BdC 15*	3000	543	180	19.00	6.43	S/1	QVVAG
Bradi2g52670	*BdC 16*	1651	411	138	14.94	5.72	S/3	QTVAG
Bradi2g58610	*BdC 17*	486	486	161	17.63	9.10	S/1	QVVSG
Bradi5g03000	*BdC 18-1*	399	399	132	14.57	8.64	?/2	QVVSG
Bradi5g03010	*BdC 18-2*	396	396	131	14.22	9.10	S/1	QYVSG
Bradi5g00460	*BdC 1-2*	378	378	125	13.14	8.6	S/2	QLVAG
Bradi5g06660	*BdC 19*	560	390	129	13.52	10.89	S/1	QIVSG

a *Localization of BdC protein supported by Target P. TargetP predictions (S: Secretary Pathways, C: cytoplasm, and ?: any other locations) and reliability class (1–5; best class is 1)*.

b *A highly conserved reactive site (QxVxG) motif presents in all cystatin proteins*.

**Figure 2:**
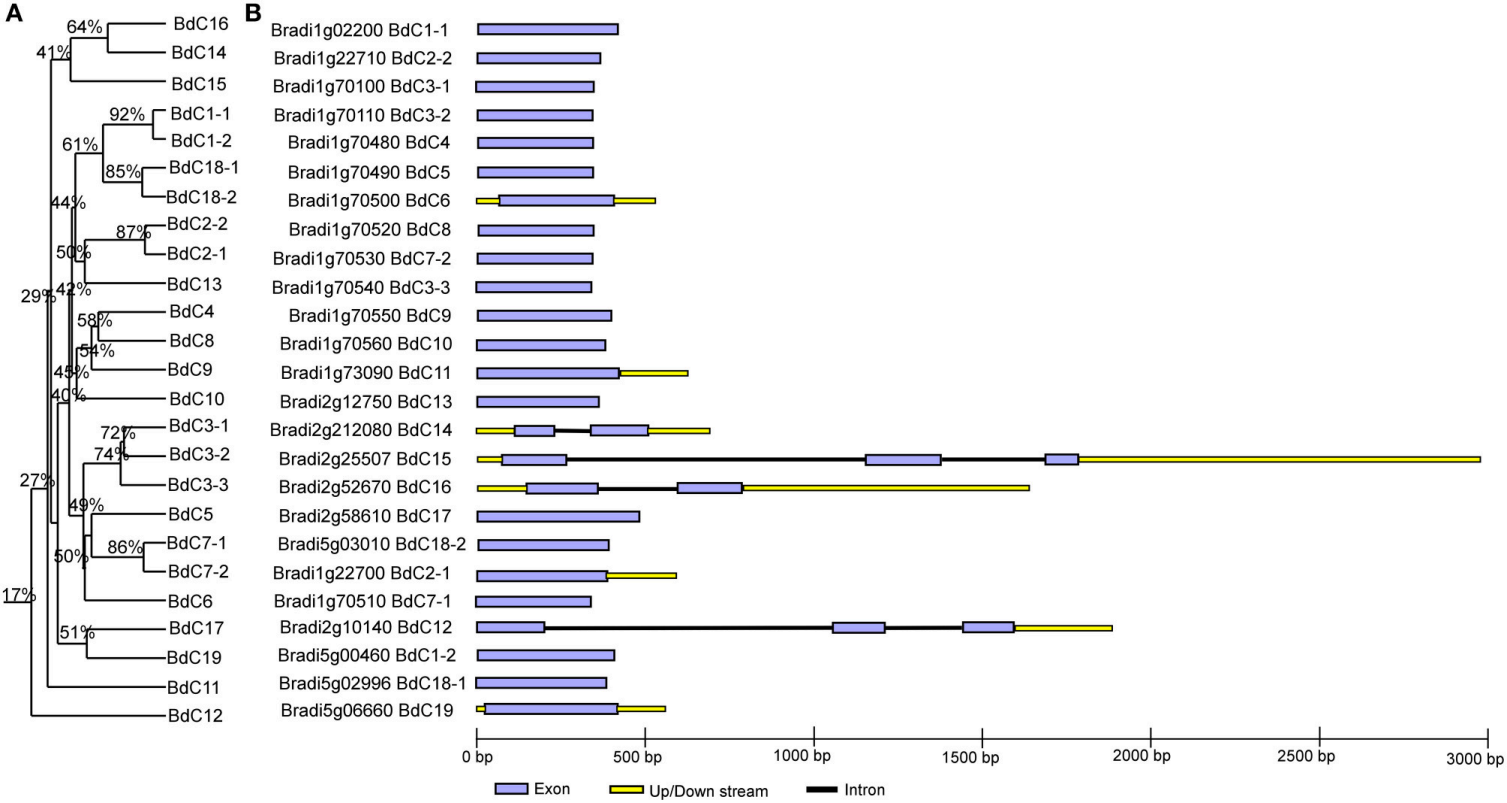
**Phylogenetic relationships and gene structure analysis of cystatin genes in *Brachypodium*. (A)** Rooted homology tree was constructed from the alignment of full-length amino acid sequences using the DNAMAN package. **(B)** Gene structure of *BdC* genes. Lavender solid boxes represent exons; black lines represent introns; yellow boxes represents up/down stream regions. Intronic phases were indicated by numbers 0, 1, and 2.

The multiple alignment of all *BdC* genes and homology tree analysis of their deduced amino acid sequences showed that 11 genes (*BdC1-1, BdC1-2, BdC2-1, BdC2-2, BdC3-1, BdC3-2, BdC3-3, BdC7-1, BdC7-2, BdC18-1*, and *BdC18-2*) belong to duplicated gene sequences. The observed sequence homology percentage of these duplicated sequences was 72–92% (Figure 2A). *BdC* genes without duplicated sequences may have originated from different progenitors. In particular, *BdC1-1* and *BdC1-2* share 92% sequence homology and are located on two different chromosomes (Nos. 1 and 5), suggesting that they may have originated by duplication of chromosomal segments. *BdC2-1* was tightly linked with *BdC2-2* on chromosome 1, sharing 87% sequence homology. Similarly, *BdC18-1* was tightly linked with *BdC18-2* on chromosome 5, and they share 85% sequence homology with each other. More interestingly, a total of 12 *BdC* genes were grouped together and are closely linked with one another at around 68 megabases (Mb) on chromosome 1 (Figure 1); however, they did not share any sequence homology except for *BdC3-1, BdC3-2*, and *BdC3-3* and *BdC7-1* and *BdC7-2*, with 74 and 86% homology, respectively.

### Gene structure and conserved motif distribution

Schematic structures of *BdC* genes were obtained using the GSDS (gene structure display server) program (Figure 2B). Average exon and intron numbers were 1.3 and 0.3, respectively. Exon numbers varied between 1 and 3, whereas intron numbers varied between 0 and 2. Only five *BdC* genes (*BdC12, BdC14, BdC15, BdC18-1*, and *BdC18-4*) consist of introns. Most *BdC* genes contained phase-0 introns and shared a similar exon/intron structure (Figure 2B). The corresponding loci and genetic characteristics of *BdC* genes are shown in Table 1. The length of the BdC proteins ranged from 112 to 180 amino acids. BdC2-2, BdC5, BdC9, BdC12, BdC13, BdC15, and BdC16 proteins (*pI* ≤ 7) were acidic, whereas the rest were basic (*pI* ≥ 7). The calculated molecular weights (kDa) and lengths (amino acids) of the open reading frames (ORF) ranged from 19.00 (*BdC15*) to 12.12 (*BdC7-1*) and from 180 (*BdC15*) to 112 (*BdC7-1*), respectively. Putative subcellular localizations of BdC proteins from a TargetP analysis were mostly in secretory pathways (Table 1).

**Figure 3:**
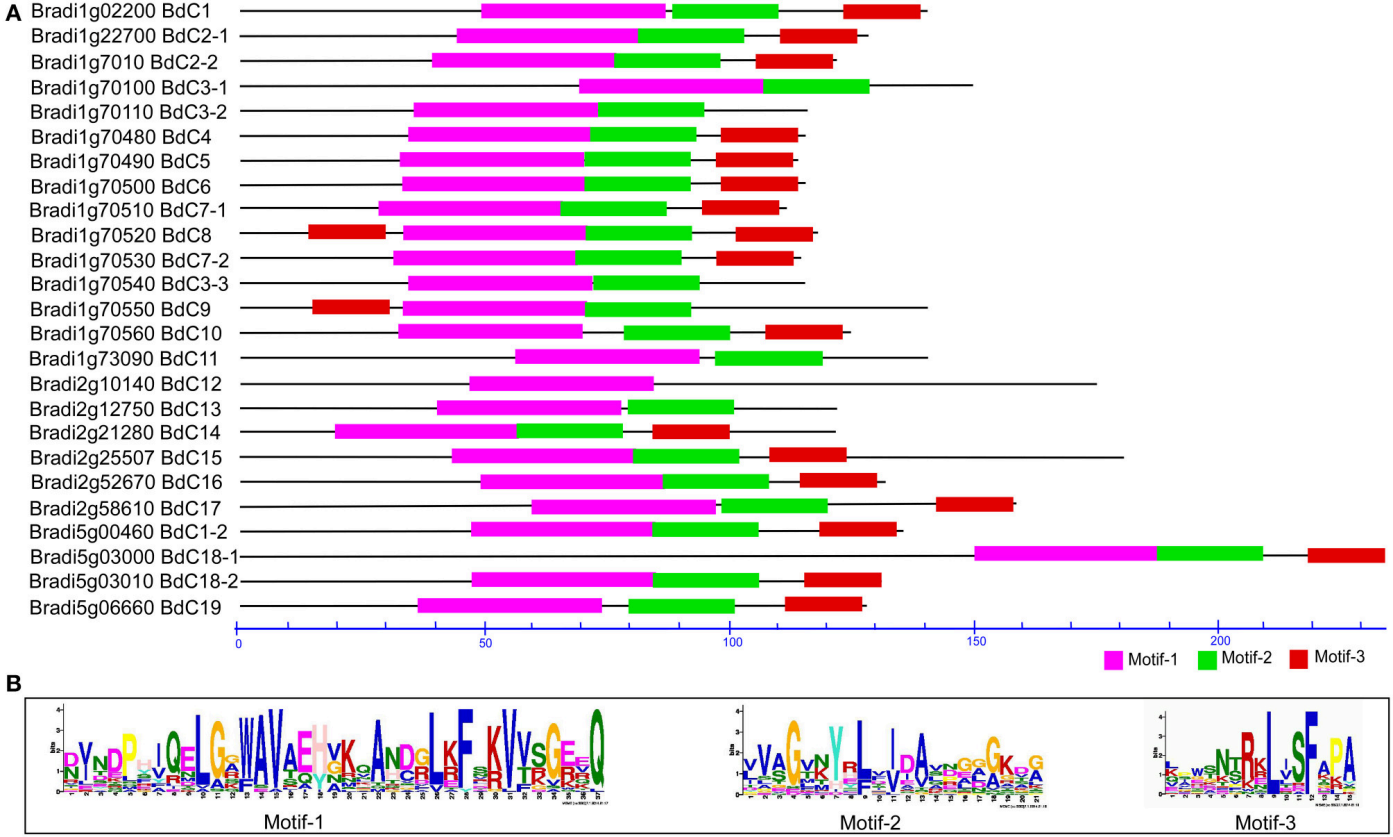
**Conserved motif analysis of BdC proteins using MEME suite (A)** Schematic diagram of amino acid motifs of cystatin genes. Motif analysis performed as described in the methods. Black solid line represents corresponding cystatin proteins and its length described by a residue scale. Various colored boxes indicating different motif and their position in each cystatin sequences as indicated. **(B)** Conserved protein motifs 1 (LARFAV), 2 (QTVAG), and 3 (W-residue) present in the variable region of cystatin genes.

In total, 71 cystatin protein sequences (Tables S1, S3) from *B. distachyon* (25), *T. aestivum* (5), *H. vulgare* (13) (Martinez et al., 2009), *S. bicolor* (1) (Li et al., 1996), *Ae. tauschii* (3) (NCBI accession: EMT22646, EMT09912, and EMT04034), *Z. mays* (10) (Massonneau et al., 2005), *O. sativa* (12) (Wang et al., 2015), *C. lacryma-jobi* (1) (Yoza et al., 2002), and *S. officinarum* (1) (Soares-Costa et al., 2002) were submitted to the MEME suite to identify conserved domains or motifs. The results showed that three common motifs were present (Figure 3A and Figure S1); of these, motifs 1, 2, and 3 form a fundamental structural combination that is present in all cystatin family members and is involved in interactions with cysteine proteinase target enzymes (Margis et al., 1998). All the predicted BdC proteins, and other cystatin proteins from various species, consist of these three motifs (Figure 3B and Figure S1). Motif 1 has a conserved N-terminal domain with a consensus sequence, L[GA]R[WF]AVAEH, that conforms to a predicted secondary α-helical structure devoid of both disulphide bonds and putative glycosylation sites [5]. Motif 2 is conserved in the central loop region and has a consensus sequence of [GA][EKR][QE]QxVxG, which acts as a reactive site. Motif 3 is conserved near the C-terminal end with a [PA]W[EL]consensus sequence, which acts as a catalytic site necessary for protein–protein interactions (Figures 3A,B).

All three motifs were observed in all the cystatin protein sequences those from different plant species except some of the cystatins for rice (OS-12) and wheat (WC-2), where motif-3 and motif-1 were missing, respectively. (Figure S1). Focusing on *Brachypodium*, motif 3 appeared twice in the BdC8 protein, once near the N-terminus and again near the C-terminus. Motif 3 was not present in BdC3-1, BdC3-2, BdC3-3, BdC11, and BdC13. Similarly, motif 2 and motif 3 were not present in BdC12 (Figure 3A). However, the rest of the predicted BdC proteins (exception) had conserved motifs 1, 2, and 3, whose locations were highly homologous in various plant species (Figure S1). The missing of motifs in several BdC proteins (BdC3-1, BdC3-2, BdC3-3, BdC11, BdC12, and BdC13) indicating a different gene structural characteristics in regard to intron-exon relationships as shown in Figure 2B. From these analyses, differences in motif distribution in cystatin proteins of plants indicated that the functions of these genes might have diverged during evolution.

### Amino acid structural analysis of *B. distachyon* cystatins

To search for amino acid variants that could lead to differences in the inhibitory capability of *B. distachyon* cystatins, alignment of all BdC sequences was performed in the CLUSTAL W program (Figure S2); this analysis included OC10 from rice, WC1 from wheat, CC1 from sorghum, and HvCPI-3 from barley. SignalP predicted the presence of signal peptides in all BdC proteins except BdC14 and BdC18-1. Cleavage site prediction was run in parallel in TargetP, thus confirming the results (Figure S2). N-terminal (BdC3-1) and C-terminal (BdC15, OC10, and HvCPI-3) extensions of varying lengths were present in several cystatins, which were not shown completely in the comparison (Figure S2). In addition to these extensions, differences in the extent of the amino acid sequences corresponding to the loops connecting the β-sheets and α-helices were also found in some cystatins, such as BdC1-1, BdC2-1, BdC8, and so on (Figure S2).

With the exception of some sequences whose signature motifs were minimally disrupted, the significant protein signatures responsible for cysteine proteinase inhibitory properties were conserved among the 25 BdC sequences. The N-terminal motif LARFAV was fairly conserved among BdC, whereas most had no perfect match with the LARFAV motif from other species (Figure S2). A hierarchical cluster analysis of BdC, along with cystatins from other species (rice, barley, wheat, and sorghum), indicated that the N-terminal G/GG was also highly conserved in BdC proteins, whereas it was absent in BdC3-1, BdC3-3, BdC5, BdC7-2, and BdC11 (Figure S3). A conserved G immediately preceded the main body in the N terminus. The region preceding the conserved G is referred to as the N-terminal trunk (NTT) and appears in some BdC proteins (Figure S3). The functionally indispensable reactive site pentapeptide sequence QXVXG was also observed in all BdC proteins based on ClustalW alignment (Figure S2) and hierarchical cluster analysis (Figure S3; Table 1). Although this site in some BdC proteins (BdC3-1, BdC3-3, BdC4, BdC5, BdC9, BdC10, BdC11, BdC12, and BdC13) was partially disrupted by various amino acid residues, this consensus sequence was almost replaced by either DPVVK (hierarchical cluster analysis) or EVVED (ClustalW alignment). Another conserved motif (P/AW) was present only near the C-terminal end in eight BdC proteins (BdC2-1, BdC2-2, BdC14, BdC15, BdC16, BdC17, BdC18-1, and BdC18-2).

To examine structural characteristics, the tertiary structure of all BdC proteins, along with WC1 (wheat), CC1 (maize), and HvCPI-3 (barley), were predicted using the Phyre2 server (Figure S4). These structures were predicted with similar degrees of accuracy, and almost all BdC proteins conserved key protein motifs with other species in the ClustalW alignment. Therefore, their tertiary structures were similar, conserving an α-helix spanning the LARFAV motif and four main β-sheets (β2, β3, β4, and β5; Figure S4). All cystatins consisted of an N-terminal α-helix along with another in the central loop region. However, two *Brachypodium* cystatins (BdC12 and Bd18-1) showed significant variations in their predicted three-dimensional structures, consisting of two α-helices in their central loop regions, probably due to two different reactive sites (QxVxG) modeled by the program (Figure S4, Table 1). The presence of amino acid residue insertions in some cystatins (BdC8, BdC9, BdC10, and BdC11) suggests that these cystatins could have a more extensive loop between each β-sheet than other cystatins. The overall predicted tertiary structures of all BdC proteins were similar to those from wheat (WC1), maize (CC1), and barley (HvCPI-3) (Figure S4).

### *BdC* promoter analysis

Generally, stress-responsive *cis*-acting elements are present in the promoter regions of stress-inducible genes. A motif search was performed using PlantCARE (Lescot et al., 2002; http://bioinformatics.psb.ugent.be/webtools/plantcare/html/) to identify putative *cis*-elements in the 1,500 bp promoter sequence upstream of the initiation codon of all *BdC* genes (Table S3). The occurrence of *cis*-elements in *BdC* genes is shown in Table 2.

**Table 2:** **Functions and number of identified *cis*-regulatory elements in *BdC* genes from *B. distachyon***.

**Motifs**	**Skn-1_motif**	**GCN4_motif**	**RY-element**	**ABRE**	**MBS**	**TC-rich repeats**	**G-Box**	**5′ UTR Py-rich stretch**	**W-box**	**HSE**	**CCAAT**
Function	*cis*-acting regulatory element required for endosperm expression	*cis*-regulatory element involved in endosperm expression	*cis*-acting regulatory element involved in seed-specific regulation	*cis*-acting element involved in abscisic acid response	MYB binding site involved in drought-inducibility	*cis*-acting element involved in defense and stress response	*cis*-acting regulatory element involved in light response	*cis*-acting element conferring high transcription levels	Wounding and pathogen responsive element	Heat shock responsive element	Enhancer- binding protein element
*BdC1-1*	1			1	1		2			1	1
*BdC1-2*	1					1	1				1
*BdC2-1*	1			1	1	1	2		1		
*BdC2-2*	1	1		1	1		2	1	1		
*BdC3-1*	1		1	1	1		2		1	1	1
*BdC3-2*	1	1	1	1	1		2	1		1	
*BdC3-3*	1	1		1	1	1	2	1	1	1	
*BdC4*	1	1				1	1		1	1	
*BdC5*	1			1	1	1	2		1		
*BdC6*	1		1	1	1		2	1			
*BdC7-1*	1	1		1	1	1	2		1		1
*BdC7-2*		1			1	1			1	1	1
*BdC8*	1	1		1	1	1	2	1	1		
*BdC9*	1			1	1	1	2	1		1	
*BdC10*		1		1	1	1	2	1		1	1
*BdC11*	1	1		1	1	1	2	1	1		1
*BdC12*	1			1			2		1	1	1
*BdC13*	1			1	1	1	2			1	
*BdC14*	1		1		1		1			1	
*BdC15*	1	1		1	1	1	2	1	1	1	
*BdC16*	1	1				1	2	1	1		
*BdC17*	1	1	1	1	1		2		1		
*BdC18-1*		1		1	1	1	2				1
*BdC18-2*	1				1		2	1		1	
*BdC19*	1	1		1	1	1	2	1	1	1	1

Several potential regulatory elements associated with stress-related transcription factor-binding sites were found, including ABA-response elements (ABREs), CCAAT boxes, heat shock elements (HSEs), stress response elements (STREs), and wound-cum-pathogen responsive elements (W-boxes) (Table 2). The CCAAT enhancer sequences represent binding sites for CCAAT enhancer binding proteins (C/EBP) and act cooperatively with HSEs to increase promoter activation under abiotic stress conditions (Rieping and Schöffl, 1992). The STRE elements are important for transcriptional activation in response to a variety of abiotic stress conditions (Siderius and Mager, 1997). The W-box (consensus sequence TTGAC) binds WRKY factors and responds to heat and wounding (Levée et al., 2004; Ülker and Somssich, 2004) and was found in almost all *BdC* genes. Similarly, ABRE is a major *cis*-acting regulatory element that plays important roles in adapting vegetative tissues to abiotic stresses, such as drought and high salinity, as well as in seed maturation and dormancy (Shinozaki et al., 2004). Three important seed-specific *cis*-motifs (Skn-1_motif, GCN4_motif, and RY-element) were conserved in the promoter regions of some *BdC* genes, suggesting that these genes are involved in regulating the gene expression of cereal grain storage proteins (Thomas and Flavell, 1990; Ueda et al., 1994). Additionally, other stress and defense responsive elements, such as TC-rich repeats, the G-Box, and the 5′UTR Py-rich stretch, were also identified among *BdC* genes. More interestingly, a light responsive *cis*-element such as G-Box was appeared two times in *BdC* promoters compare to other *cis*-elements, suggesting that *BdC* genes may highly inducible by light stress.

### Phylogenetic analysis of *Brachypodium* cystatins

**Figure 4:**
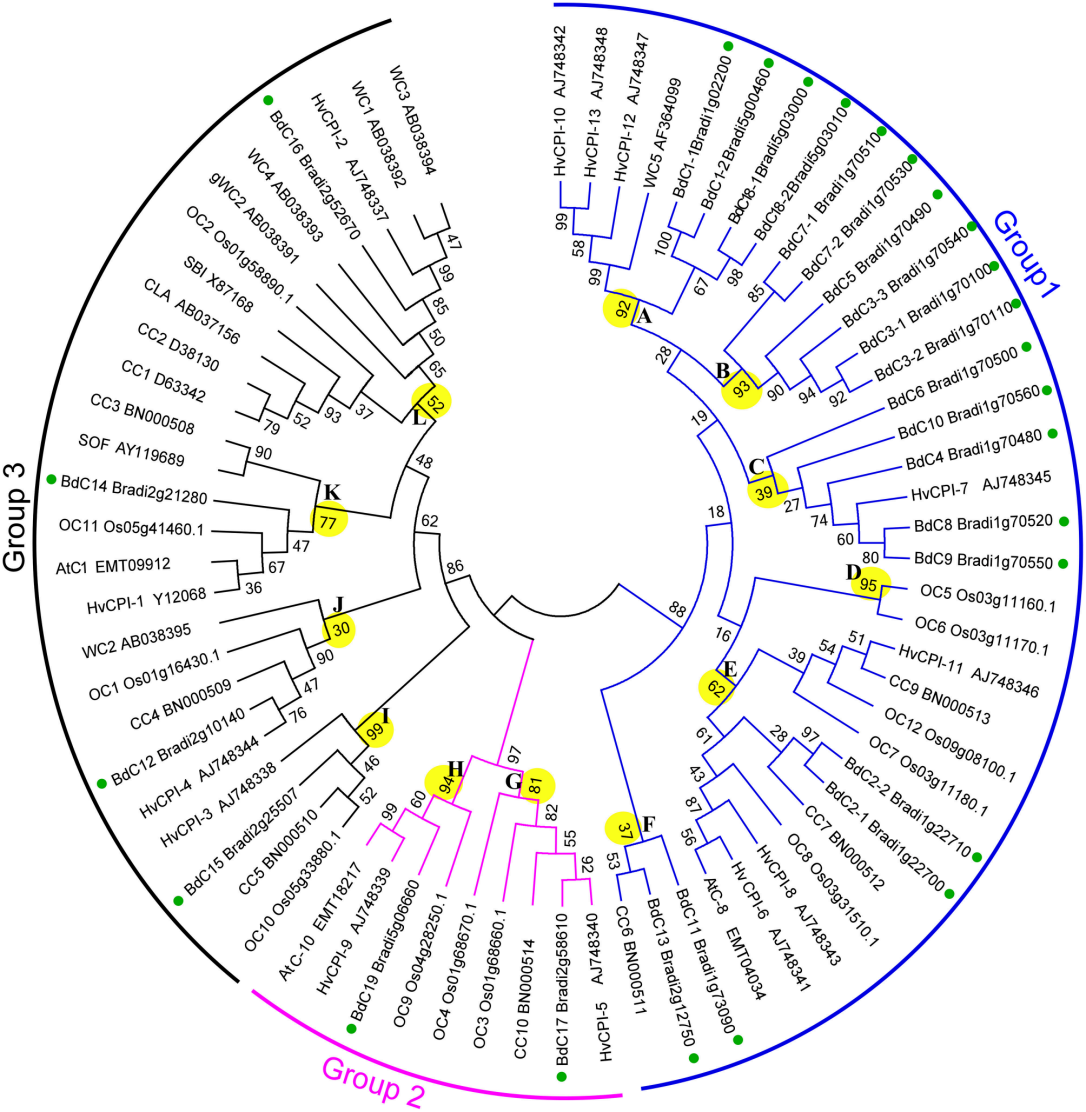
**Phylogenetic tree of the Poaceae cystatins showing relationships between the deduced amino acid sequences of 71 cystatin genes from different plant species**. 25 from *B. distachyon* (BdC), 5 from *T. aestivum* (WC), 13 from *H. vulgare* (HvCPI), 1 from *S. bicolor* (SbC), 3 from *Ae. tauschii* (AeC), 10 from *Z. mays* (CC), 12 from *O. sativa* (OC), 1 from *C. lacryma-jobi* (CLA) and 1 from *S. officinarum* (SOF). Multiple alignments of sequences were performed by ClusalW, and the phylogenetic tree was constructed by the neighbor-joining (NJ) method and evaluated by bootstrap analysis. Numbers on the main branches indicate boot strap percentages for 1,000 replicates. The three major groups (1–3) and twelve phylogenetic subgroups (A–L) identified in the plant cystatin family are highlighted with different color arcs and branch, respectively. GenBank numbers for corresponding to the sequences are also shown. Green full circles indicate the *BdC* genes in the branches.

To investigate the phylogenic relationships of cystatin genes from *B. distachyon* and eight other Poaceae species and to generate an evolutionary framework, an unrooted phylogenetic tree was constructed from an alignment of 71 cystatin amino acid sequences (Tables S1, S3), including 25 from *B. distachyon* (BdC), 5 from *T. aestivum* (WC), 13 from *H. vulgare* (HvCPI), 1 from *S. bicolor* (SbC), 3 from *Ae. tauschii* (AeC), 10 from *Z. mays* (CC), 12 from *O. sativa* (OC), 1 from *C. lacryma-jobi* (CLA), and 1 from *S. officinarum* (SOF). As shown in Figure 4, three major groups (1, 2, and 3) and 12 phylogenetic subgroups (A–L) were clearly present among these cystatins. The bootstrap values for the major groups ranged from 86 to 97%, indicating strong support for their phylogenetic relationships. The largest group, group 1, containing 36 cystatins from BdC, HvCPI, WC, OC, CC, and AeC, was divided into subgroups A–F. The smallest group, group 2, was separated into subgroups G–H and consisted of nine cystatins from BdC, OC, AeC, and HvCPI. Group 3 was divided into subgroups I–L and contained 26 cystatins from all Poaceae species investigated. Among the 25 *Brachypodium* cystatins, 19, 2, and 4 were distributed in groups 1, 2, and 3, respectively. Generally, *Brachypodium* cystatins were close to those from barley (HvCPI), wheat (WC), and maize (CC).

### Differential mRNA expression of *BdC* genes in different organs and determining cDNA copy numbers of duplicated *BdC* genes

**Figure 5:**
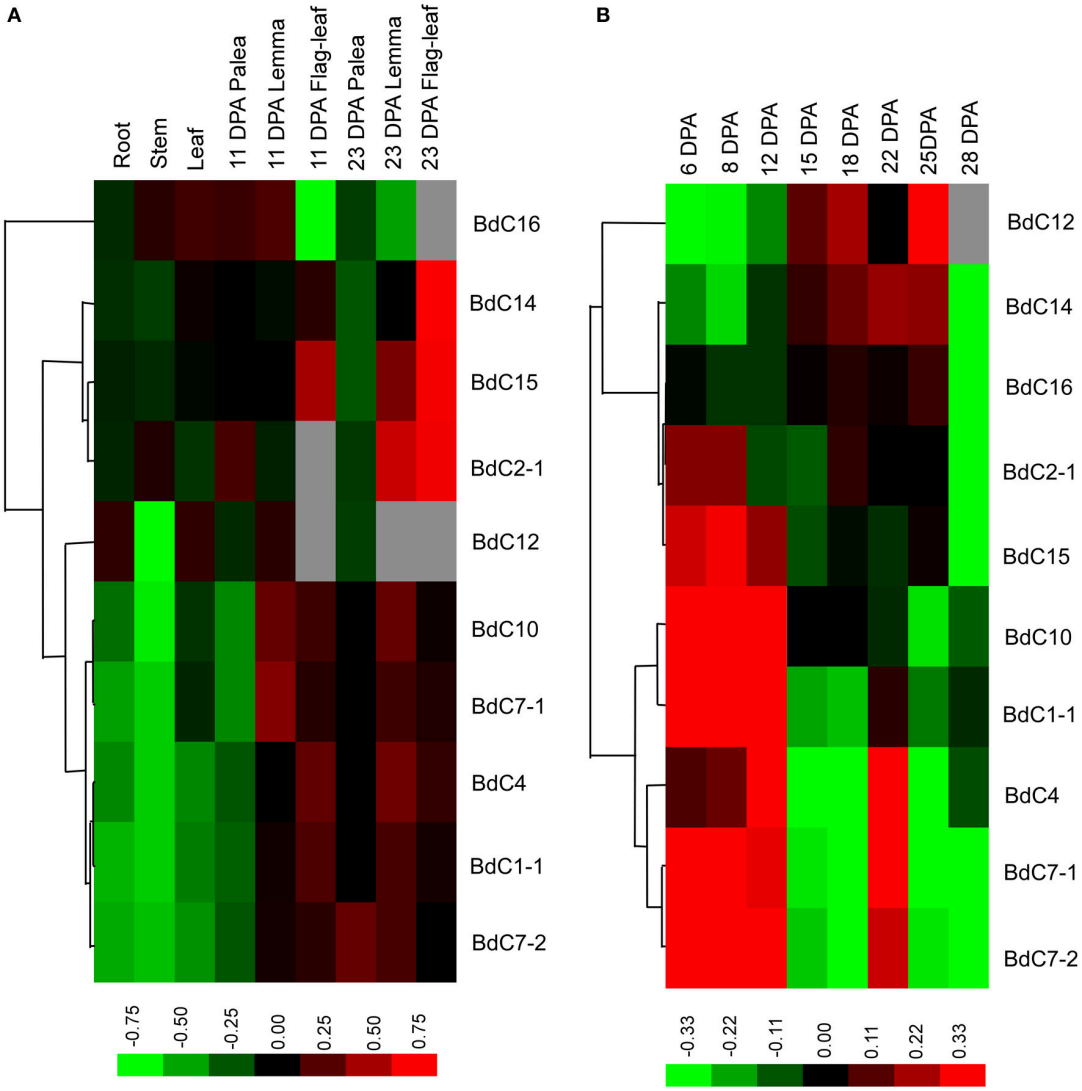
**Expression profiling of *BdC* genes in different *B. distachyon* organs detected by qRT-PCR. (A)** Comparative expression levels of 10 *BdC* genes in different *Brachypodium distachyon* organs, including roots, stems and leaves at the two-leaf stage; seed palea, lemma, flag-leaf at 11, and 23 days after anthesis (DPA). **(B)** Dynamic expression profiles of 10 *BdC* genes during seed development in Bd21. Relative quantification of the expression levels in developing caryopses was collected between 6 and 28 DPA. Log transform data was used to create the heatmap. Expression data were obtained from three biological replicates. Differences in gene expression changes are shown in color as the scale.

The transcriptional expression levels of 10 typical *BdC* genes in different organs, including roots, stems, and leaves at the two-leaf and heading stages, paleas, lemmas, seeds, and developing caryopses, were investigated using qRT-PCR (Figure 5). Specific primer sets were designed for each *BdC* gene (Table S2). *BdC1-1, BdC4, BdC7-1, BdC7-2*, and *BdC10* had lower expression levels in roots, stems, leaves from seedlings at the two-leaf stage, and 11 DPA paleas compared with *BdC2-1, BdC12, BdC14, BdC15*, and *BdC16*, whereas the expression of *BdC12* was abundant in roots, leaves, and 11 DPA lemmas. *BdC2-1, BdC14*, and *BdC15* displayed higher expression levels in lemmas (23 DPA) and flag-leaves (23 DPA) compared with other organs. Two pairs of *BdC* genes (*BdC1-1* and *BdC4, BdC7-1*, and *BdC10*) shared similar patterns of expression. In contrast, *BdC7-1* and *BdC7-2*, originating from a duplication event, had significantly different expressions in 23 DPA paleas (Figure 5A). Ten *BdC* genes displayed distinct expression profiles in different organs.

The dynamic transcription expression levels of 10 *BdC* genes in 8 different grain developmental stages were investigated (Figure 5B). The sampled caryopses covered the main stages of endosperm development from cellularization to desiccation. These 10 *BdC* genes displayed three main expression patterns. The first pattern was displayed by three *BdC* genes (*BdC12, BdC14*, and *BdC16*) and showed gradually increasing expression levels from 6 to 15 DPA, reaching the highest level between 18 and 25 DPA, and decreasing until 28 DPA. The second pattern, displayed by four *BdC* genes (*BdC1-1 BdC10, BdC2-1*, and *BdC15*), had high expression levels in early grain developmental stages (6–12 DPA) and then decreased and were maintained at relatively low abundances. The remaining genes (*BdC4, BdC7-1*, and *BdC7-2*) displayed a third expression pattern, with much higher abundances in the early grain developmental stages (6–12 DPA); these levels then decreased slightly or remained relatively low until 18 DPA, after which expression levels increased dramatically, reaching a peak at 22 DPA. It should be noted that both *BdC7-1* and *BdC7-2* had a uniform expression pattern, with higher expression in developing seeds than in vegetative organs.

Real-time quantitative PCR has been proved to be an efficient method for the quantification of cDNA copy numbers of gene transcripts in which the presence of alleles of large gene families with highly homologs members could be detected in a cDNA population by discriminate the expression level between genes or individual members (d'Aloisio et al., 2010; Kaczmarczyk et al., 2012). In the present study, we found several tandem or segmentally duplicated genes which were shown high allelic similarities to their corresponding duplicated gene pair during sequence comparisons (Figure 2A). In order to confirm the existence of duplicated *BdC* genes in cDNA population through discriminate the expression levels between the individual *BdC* members, we carried out an absolute mRNA expression analysis and determined the number of cDNA copies of several tandem (*BdC3-1, BdC3-2, BdC3-3*) and segmentally duplicated (*BdC1-1* and *BdC1-2*) genes. Allelic specific primers were designed to discriminate between the duplicated *BdC* members to prove the primer specificity on the cDNA template of the corresponding allele (Table S2). Among three different tissues (leaf, root and seed) were screened by absolute qRT-PCR analysis, *BdC1-1* showed the highest amount of cDNA copies in leaf (6.18 × 10^2^) where *BdC1-2* had about 4.27 × 10^2^. Similarly, a maximum number of *BdC1-2* cDNA copies was estimated as 7.50 × 10^0^ at seed where *BdC1-1* were only 2.45 × 10^1^, as shown in Table S4. While comparing the amount of cDNA copies among *BdC3* members in three different tissues, *BdC3-1* had a maximum number of cDNA copies at seed (9.20 × 10^2^) which was higher than cDNA copies of either *BdC3-2* (2.73 × 10^1^) or *BdC3-3* (1.18 × 10^4^). However, *BdC3-2* in root exhibited a higher level of cDNA copies (8.17 × 10^0^) where *BdC3*-1 and *BdC3-3* possessed only 2.68 × 10^1^ and 3.43 × 10^5^, respectively. Similar to root and seed, leaf tissue also showed a considerable variation in amount of cDNA copies between *BdC3-1* (1.58 × 10^1^), *BdC3-2* (1.07 × 10^2^) and *BdC3-3* (2.20 × 10^6^) as shown in Table S4. The observed substantial fluctuation of transcription rates between these genes in each tissues might adequate to discriminate their expression levels and thus confirming that there are duplicated *BdC* genes in cDNA population; furthermore the presence of duplicated *BdC* genes in cDNA population may provided a suggestive evidence for the existence of duplicated copies of *BdC* genes in *Brachypodium* genome.

### Expression profiling of *BdC* genes under various abiotic stresses

**Figure 6:**
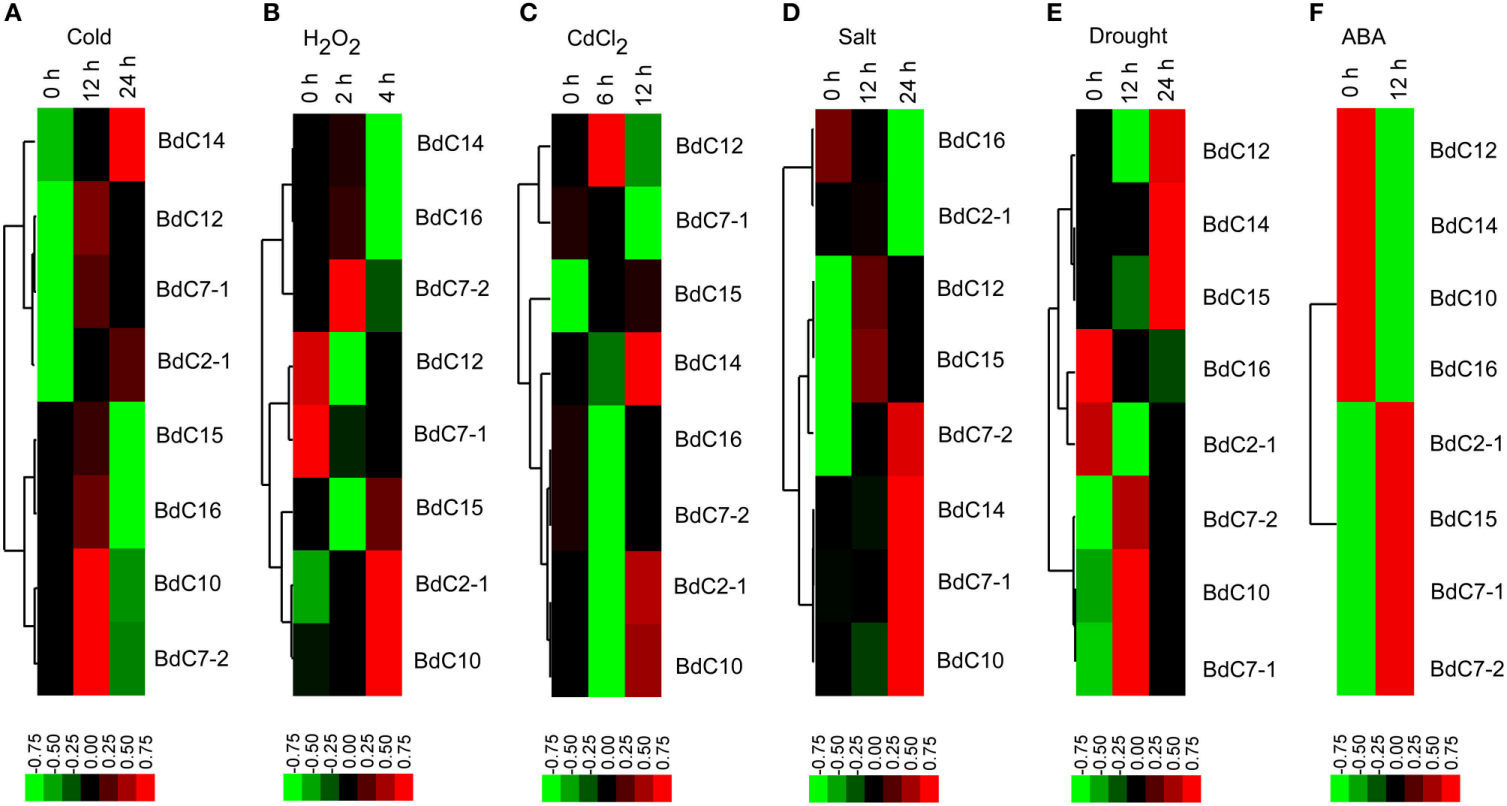
**Expression profiles of *BdC* family members in the leaves of *B. distachyon* in response to different abiotic stress treatments (cold, H_2_O_2_, CdCl_2_, drought, salt, and ABA) by qRT-PCR**. Blocks with colors indicated decreased (green) or increased (red) transcript accumulation relative to the respective control. Filled squares indicate a significant difference from the control (*p* < 0.05) by using SPSS (Statistical Product and Service Solutions) software. Expression profiles of the *BdC* genes under cold **(A)**, H_2_O_2_ **(B)**, CdCl_2_ **(C)**, drought **(D)**, salt **(E)** and ABA **(F)** stresses from different time points are indicated.

The expression profiles of eight representative *BdC* genes under six abiotic stresses (cold, H_2_O_2_,CdCl_2_, salt, drought, and abscisic acid (ABA) are shown in Figure 6. Under cold stress, most *BdC* genes displayed up-then-down regulated expression patterns from 0 to 24 h. Only two genes (*BdC14* and *BdC2-1*) were continuously upregulated until 24 h (Figure 6A). Four *BdC* genes (*BdC7-2, BdC2-1, BdC10*, and *BdC15*) were specifically upregulated at 2 h or 4 h under H_2_O_2_ treatment, and two genes (*BdC7-1* and *BdC12*) were most highly expressed only under normal conditions (Figure 6B). Under CdCl_2_ stress, *BdC12* and *BdC15* were significantly upregulated at 6 h, whereas three genes (*BdC2-1, BdC10*, and *BdC14*) were significantly upregulated at 12 h (Figure 6C). *BdC12* and *BdC15* were upregulated at 12 h under salt stress, whereas four (*BdC7-1, BdC 7-2, BdC10*, and *BdC14*) and two (*BdC2-1* and *BdC16*) *BdC* genes were upregulated and downregulated under 24 h of salinity stress, respectively (Figure 6D). Under drought stress induced by PEG 6000, several *BdC* genes were specifically upregulated only at 12 h (*BdC7-1, BdC7-2*, and *BdC10*) or 24 h of treatment (*BdC12, BdC14*, and *BdC15*; Figure 6E). The expression patterns under abscisic acid (ABA) treatment showed that *BdC2-1, BdC7-1, BdC7-2*, and *BdC15* were upregulated, whereas *BdC10, BdC12, BdC14*, and *BdC16* were downregulated (Figure 6F).

In general, all *BdC* genes were upregulated in response to two or more stresses. *BdC15* was upregulated at different levels under all stresses. *BdC7-2* and *BdC10* were upregulated under five stresses, but it was downregulated and slightly downregulated by CdCl_2_ and ABA stress, respectively (Figures 6A–F). Under four stresses, *BdC2-1 BdC7-1, BdC12*, and *BdC14* were upregulated, whereas they were downregulated in response to H_2_O_2_ stress (Figures 6A–F). Moreover, *BdC14* and *BdC12* were downregulated under ABA stress, whereas *BdC7-1* was downregulated under CdCl_2_ stress (Figures 6C,F). *BdC16* was specifically upregulated only by the following two stresses: cold and H_2_O_2_ (Figures 6A,B, 6D–F).

## Discussion

### Evolutionary conservation and divergence of the *cystatin* gene family in *B. distachyon*

Our results revealed that *Brachypodium distachyon BdC* genes were unevenly distributed on chromosomes 1, 2, and 5, and chromosome 1 contained the highest *BdC* density, followed by chromosomes 2 and 5 (Figure 1). More than half were distributed on chromosome 1, suggesting that cystatin genes in *Brachypodium distachyon* may have a chromosomal preference. Phylogenetic analysis showed that *BdC* genes, as well as those from eight other Poaceae species, were separated into three well-conserved groups. Most *BdC* genes shared similar exon/intron structure and motif organization, suggesting that *BdC* genes maintained high structural conservation over a long evolutionary process. Additionally, the phylogenetic tree also showed that the genetic relationships of *Brachypodium* cystatins were much closer to barley, wheat, and maize than to other species, such as rice, Job's tears, sugarcane, and *Aegilops*, as reported previously (International Brachypodium Initiative, 2010; Brenchley et al., 2012).

Open reading frames of different sizes and partial motif deletions and mutations of some important amino acids showed that the *Brachypodium BdC* gene family probably underwent a complex evolutionary history, involving unequal recombination, duplication, and deletion of gene fragments. These changes would have a significant influence on their respective functions (Kondo et al., 1990; Abraham et al., 2006). We found that some BdC proteins and the QxVxG active site motif in the central loop region were partially (BdC1, BdC3-1, BdC3-3, BdC5, BdC9, BdC10, BdC11, and BdC13) or completely (BdC4 and BdC12) modified by the insertion of or variation in important residues (Table 1). Similar variations in the QxVxG site and its altered inhibitory action against cysteine proteinase were reported previously (Melo et al., 2003). Additionally, the presence of NTT and W residues (near the C-terminal region) may interact with cysteine proteases (Neuteboom et al., 2009) and actively participate in the inhibition of papain, cathepsin B, or cathepsin H, antifungal activities reported in a previous analysis (Abraham et al., 2006). We speculated that some hypervariable sites may be located at strategic positions on the protein: on each side of the conserved glycine residues in the NTT, within the first and second inhibitory loops entering the active site of target enzymes, and surrounding the LARFAV motif; these were assumed to be positively selected and thus implicated in functional diversity (Kiggundu et al., 2006). However, whatever variations are present at the structural level of *BdC* genes, the basic 3D structural fold comprising an α-helix and at least four antiparallel β-sheets (β1, β2, β3, and β4) that clearly distinguishes them as cystatins were conserved in the *BdC* gene structure, as reported in rice and barely (Abraham et al., 2006; Figure S4).

### *BdC* gene expression and plant growth and grain development

As revealed by qRT-PCR, the transcript expression levels of *BdC* family members in six different tissues confirmed that they were not organ-specific, consistent with previous reports (Kuroda et al., 2001; Abraham et al., 2006; Valdes-Rodriguez et al., 2007). In the present study, several *BdC* genes, such as *BdC2-1, BdC14*, and *BdC15*, had higher expression levels in 23 DPA flag-leaves (Figure 5A), whereas others (*BdC12* and *BdC16*) had significant mRNA accumulation in lemmas (11 DPA and 23 DPA). Additionally, *BdC2-1* and *BdC16* were considerably more highly expressed than other *BdC* genes in stem vegetative tissues (Figure 5A). These results indicate that *BdC* genes are differentially expressed, implying they play specific roles in different organs or stages of plant growth and development.

Dynamic transcript expression profiling of *BdC* genes during seed development (Figure 5B) demonstrated that almost all *BdC* genes (except *BdC12, BdC14*, and *BdC16*) had much higher expression levels during early seed development (6–12 DPA), which is similar to results from rice caryopse formation, in which two distinct oryzacystatin (OCI and OCII) mRNAs could be detected as early as 2 weeks after pollination (Abe et al., 1987). Similarly, some wheat cystatin (WC1, WC2, and WC4) mRNAs were detected in seeds only during the first 2 weeks after pollination (Kuroda et al., 2001; Corre-Menguy et al., 2002). In addition to the early grain developmental stages, the expression of some *BdC* members (*BdC4, BdC7-1, BdC7-2, BdC12*, and *BdC14*) were detected between 18 and 25 DPA (Figure 5B), corresponding to the mRNA transcript accumulation of corn cystatin (CC) in late caryopsis development, between 15 and 30 DPA (Arai et al., 2002). In general, most *BdC* genes are specifically expressed in developing seeds and contain the *cis*-acting regulatory elements required for endosperm expression (Table 2). According to previous reports, phy-cys in seeds can play different roles, including regulation of protein turn-over during seed maturation (Kiyosaki et al., 2007), control of proteolysis during development and/or germination (Gaddour et al., 2001), and protection of seeds against pests (Martinez et al., 2009).

In wheat, WC5 mRNA accumulation was observed only in grain tissues, and its inhibitory action against thiol peptidase from seed protein extracts suggests that seed-specific cystatins play important roles as regulators of peptidase enzymes during seed development (Corre-Menguy et al., 2002). Barley cystatins (Icy1, Icy2, Icy3, and Icy4), primarily cathepsin L-like cys-proteases, were shown to be preferentially expressed in dry and germinating seeds and were efficient inhibitors. This suggests that their main roles are as specialized endogenous regulators of enzymes involved in the mobilization of stored proteins upon germination, which is crucial for seedling growth until photosynthesis is fully established (Martinez et al., 2009). Similarly, some rice and wheat cystatins were also shown to inhibit cys-proteases, such as oryzains and gliadains, which are involved in turnover functions in rice and wheat aleurone layers, respectively (Arai et al., 2002; Kiyosaki et al., 2007). Therefore, further experimental studies are necessary to elucidate the functional role of *BdC* members in the mobilization of storage reserves and their inhibitory action against proteases in *Brachypodium* seeds.

### Expression profiling and potential functions of *BdC* genes in response to different abiotic stresses

*Cystatin* genes are implicated in various abiotic stress responses in different plant species, including *Arabidopsis thaliana* (Zhang et al., 2008), chestnut (Pernas et al., 2000), barley (Gaddour et al., 2001), cowpea (Diop et al., 2004), maize (Massonneau et al., 2005), and rice (Huang et al., 2007). In the present study, all stress treatments (cold, H_2_O_2_, CdCl_2_, salt, drought, and ABA) induced strong *BdC* accumulation in leaves, suggesting that *BdC* genes are involved in the stress-responsive mechanism of *Brachypodium* plants. Among upregulated *BdC* genes, six (*BdC7-1, BdC7-2, BdC10, BdC12, BdC14*, and *BdC15*) were upregulated in response to more than three stress treatments (Figure 6). Furthermore, promoter analysis showed that three stress-related *cis*-elements (ABRE, MBS, and TC-rich repeats) that frequently occur in the promoter regions of abiotic stress defense pathway genes were also present in the promoter region of these *cystatin* genes (Table 2). These results imply that *BdC* genes could be involved in multiple stress defense mechanisms, as reported previously (Zhang et al., 2008). Different *BdC* genes exhibited differential expression patterns in response to six different stress treatments (Figure 6), indicating that functional differentiation among these *BdC* genes occurred during the evolutionary process.

In general, adverse conditions, such as salt, drought, abscisic acid, and H_2_O_2_, often result in the accumulation of reactive oxygen species (ROS) in plant cells, which can change the structural properties of proteins (Berlett and Stadtman, 1997). This can lead to the accumulation of un-folded or mis-folded aberrant proteins, which are degraded mostly by cysteine protease (Demirevska et al., 2010). To maintain optimum protein degradation by cysteine protease, plants synthesize protease inhibitors, such as cystatins, to regulate cysteine protease activities under stress conditions (Zhang et al., 2008). Therefore, *BdC* members participate in the regulation of protease enzymes, enhancing the tolerance of *Brachypodium* to abiotic stress.

Upregulated expression of *BdC2-1, BdC7-2, BdC10*, and *BdC15* under H_2_O_2_ indicated that these genes are involved in antioxidant defense. A signal pathway that leads to PCD is initiated and spread via increasing accumulation of ROS induced by salt and H_2_O_2_ (Desikan et al., 1998; Belenghi et al., 2003). ROS-triggered PCD is regulated by cysteine proteases, which play an instrumental role in this physiological process (Solomon et al., 1999). Plants can control PCD by inhibiting the activity of cysteine proteases by regulating the expression of specific protease inhibitor genes. In *Brachypodium*, the upregulation of *BdC* genes induced by H_2_O_2_ treatment acts as an inhibitor to regulate the activity of cysteine proteases, suppress PCD, and enhance plant antioxidant defense.

Differential transcriptional induction of genes is often influenced by the presence or absence of *cis*-regulatory elements in the promoter region. In addition to ABRE, MBS, and TC-rich repeats, we also observed the presence of other abiotic stress responsive *cis*-elements, such as the G-box (light responsive motif), W-box (wound and pathogen response), and HSE (heat stress). As shown in Table 2, G-box is the most prevalent *cis*- regulatory element motif presented in *BdC* genes, and their functional implications in light and other abiotic stresses already have been described well in a previous report (Qin et al., 2011). G-box was considered as the cognate *cis*-element for the basic zipper (bZIP) (de Pater et al., 1993) or basic helix–loop–helix (bHLH) transcription factors (TF; Kawagoe and Mura, 1996). In rice, G-box elements were reported to be significantly enriched in promoter regions of upregulated senescence-inducible genes in response to various hormonal stress (abscisic acid, brassinosteroid, JA and GA) where TFs (bHLH and bZIP) were shown to highly express, further suggesting that G-box elements were effectively involves in inducibility of senescence under *in vivo* and *in vitro* conditions (Liu et al., 2016a). In Arabidopsis, *PSEUDO-RESPONSE REGULATORs* (PRRs) act as transcriptional repressors and play important roles in regulating flowering time and abiotic stress responses. Here, G-box like motifs were observed to be overrepresented in PRRs and showed to be an important *cis*- regulatory element for mediating the transcriptional regulation of *CIRCADIAN CLOCK ASSOCIATED 1* (CCA1) by PRRs (Liu et al., 2016b). These observations provide an important foundation for further functional studies of *BdC* genes. Some studies also reported the expression of cystatins in roots, shoots (Christova et al., 2006), and stems (Valdes-Rodriguez et al., 2007), and the expression profiling of *BdC* genes in other tissues under various biotic and abiotic stress treatments awaits further research. The abiotic-stress-induced expression of *BdC* genes may contribute to the regulation of PCD triggered in *Brachypodium* in response to unfavorable growth conditions, as reported in other model plant systems (Solomon et al., 1999; Belenghi et al., 2003).

### A putative cystatin gene pathway in response to abiotic stress

**Figure 7:**
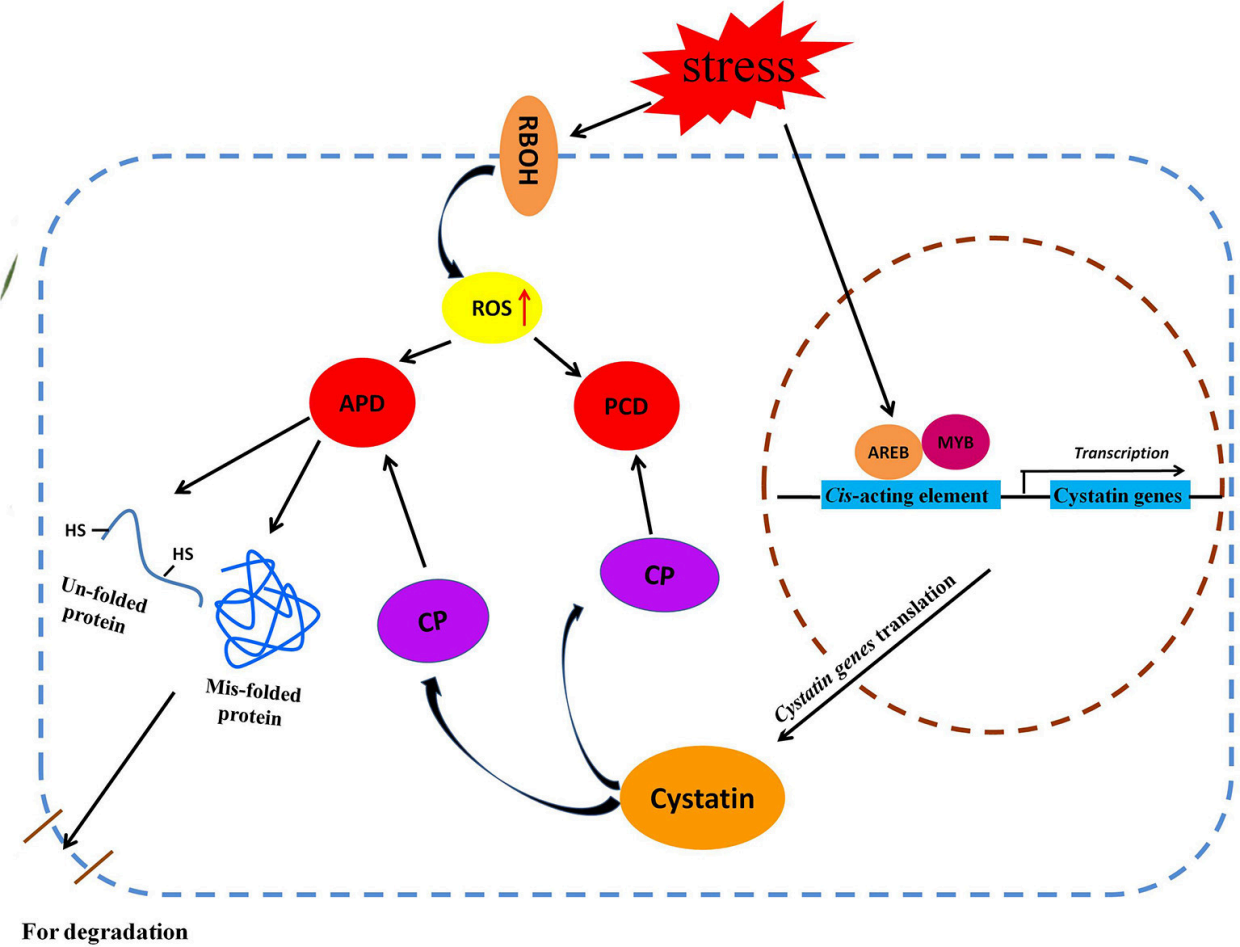
**Schematic representation of putative metabolic pathways of cystatin genes involved in two major metabolic pathway (APD and PCD) under various abiotic stresses**. AREB, ABA-responsive element binding protein; APD, Aberrant protein degradation; CP, Cysteine protease; MYB, Myeloblastosis family of transcription factor; PCD, Programmed cell death; RBOH, Respiratory burst oxidase homolog.

Based on our results, and in combination with previous reports, we propose a putative metabolic pathway for cytastin genes in response to various abiotic stresses; this pathway mainly involves the aberrant protein degradative pathway and the ROS-triggered PCD signaling pathway (Figure 7). Abiotic stresses, such as salt, drought, abscisic acid, and H_2_O_2_, induce accumulation of ROS in plant cells, which affects the structural properties of proteins (Berlett and Stadtman, 1997). This may result in the generation of un-folded and mis-folded aberrant proteins that are degraded by cysteine proteases (Demirevska et al., 2010). To maintain optimum protein degradation, cytastins are synthesized to regulate cysteine protease activity in response to various abiotic stresses (Zhang et al., 2008). Meanwhile, the ROS-triggered PCD signaling pathway is activated by the increasing accumulation of endogenous ROS (Desikan et al., 1998; Belenghi et al., 2003). The ROS-triggered PCD is regulated by cysteine proteases, which play an instrumental role in this physiological process. To prevent unwanted cell death, plants upregulate the expression of *cytastin* genes to inhibit the activity of cysteine proteases, indirectly controlling the PCD process (Zhang et al., 2008). Under various abiotic stresses, *BdC* genes, regulated by stress-related *cis*-acting elements present in the promoter region, participate in these signaling pathways by regulating the activities of cysteine proteases.

## Conclusion

In the current study, we identified 25 *BdC* genes in the *B. distachyon* genome through *in silico* analysis. All *BdC* genes shared similar exon/intron organization with three conserved motifs, which is similar to those from other plant species. Phylogenetic analysis revealed that *BdC* genes were highly orthologous to those from barley, wheat, and maize. Variations in genomic organization, deletions in motifs, and mutations in critical active site amino acids suggest that these genes underwent a complex evolutionary process and structural and functional divergence. The differential expression patterns in developing caryopses and under various abiotic stress conditions revealed that *BdC* genes involved in the regulation of cysteine protease activity could mobilize storage reserves and play crucial roles in the response to multiple abiotic stresses through the degradation of aberrant proteins and the ROS-triggered PCD signaling pathway. These results provide a better understanding of the structure and function of the *BdC* gene family.

## Author contributions

SS and DZ carried out the experiments and drafted the manuscript. XL and YH participated in the study and helped to draft the manuscript. YY conceived the study, planned experiments, and helped draft the manuscript. All authors have read and approved the final manuscript.

### Conflict of interest statement

The authors declare that the research was conducted in the absence of any commercial or financial relationships that could be construed as a potential conflict of interest.

## Supplementary material

Figure S1 **Motifs in cystatin proteins from different plant species identified by MEME analysis**. Different colored boxes indicate different motifs and their positions in each cystatin sequence.

Figure S2 **Amino acid clustalW multiple alignment of the 25 members of *Brachypodium distachyon* cystatins (BdC) along with barely (HvCPI-3), wheat (WC1), rice (OC10) and sorghum (CC1)**. The locations of the secondary structures (α-helix and β-sheets) are included. Signal peptides are marked by blue shades. The conserved signature sequences of the phy-cys are highlighted by enclosing in colored rectangles (Black, N-terminal G; Blue, LARFAV; Red, QXVXG; Pink, P/AW). Signal peptides are underlined. Few *N*-terminal (BdC3-1, BdC18-1) and *C*-terminal (BdC15, OC10, and HvCPI-3) amino acid residues are not shown here.

Figure S3 **Hierarchical clustering of cystatins from *Brachypodium* along with related species**. The conserved signature motifs are indicated by colored rectangles (Blue, LARFAV; Red, QXVXG; Pink, P/AW). The yellow shaded residues are claimed to be the putative N-terminal “G” residues. The predicted consensus sequences are represented by an orange color box.

Figure S4 **Three dimensional structures of *B. distachyon* BdC proteins**. The tertiary structure was predicted by the Phyre2 server and structure composition indicated similarity with the structure of barely (HvCPI-3), wheat (WC1), and sorghum (CC1) as indicated. The secondary structure is shown with α helixes in green, β sheets in yellow, and loops in blue.

Table S1 **List of cystatin amino acid sequences from various plant species used in this study**.

Table S2 **List of Primers designed and used for qRT-PCR analysis of mRNA expression of *cystatin* genes from *B. distachyon* (*BdC*)**.

Table S3 **List of identified *BdC* gene models and their sequence information on *B. distachyon***.

Table S4 **Absolute quantification of the mRNA expression level of duplicated *BdC* genes**. Quantification was according to the reference gene (*Ubiquitin*) in the quantitative real-time polymerase chain reaction (qRT-PCR) analysis. The values listed are indicated as cDNA copies/μg of reverse-transcribed total RNA, and the data are shown as the mean ± standard deviation.

## Abbreviations

ABA: abscisic acid
ABRE: ABA-response element
AREB: ABRE-binding protein
ABRE: ABA-responsive element
APD: Aberrant proteins degradation
C/EBP: CCAAT enhancer binding proteins
CDS: Coding sequences
CP: Cysteine protease
DPA: Days post-anthesis
GSDS: Gene structure display server
G-Box: Light responsive motif
HSE: Heat shock element
MBS: MYB binding site
MYB: Myeloblastosis family of transcription factor
MW: Molecular weight
MEME: Multiple Em for Motif Elicitation
MEGA: Molecular Evolutionary Genetic Analysis
NJ: Neighbor- joining
NTT: N-terminal trunk
ORF: Open reading frame
PCD: Programmed cell dead
PGDD: Plant Genome Duplication Database
PFAM: Programmed frequency amplitude modulation
PCR: Polymerase Chain Reaction
qRT-PCR: Quantitative Real-time PCR
ROS: Reactive oxygen species
RBOH: Respiratory burst oxidase homolog
STRE: Stress response element
W-boxe: Wound cum pathogen responsive element.
