# Supplementary figures and images for: Molecular Characterization and Expression Profiling of Brachypodium distachyon L. Cystatin Genes Reveal High Evolutionary Conservation and Functional Divergence in Response to Abiotic Stress
Source: Front Plant Sci. 2017 May 9;8:743. doi: 10.3389/fpls.2017.00743 (PMC5423411; doi:10.3389/fpls.2017.00743)

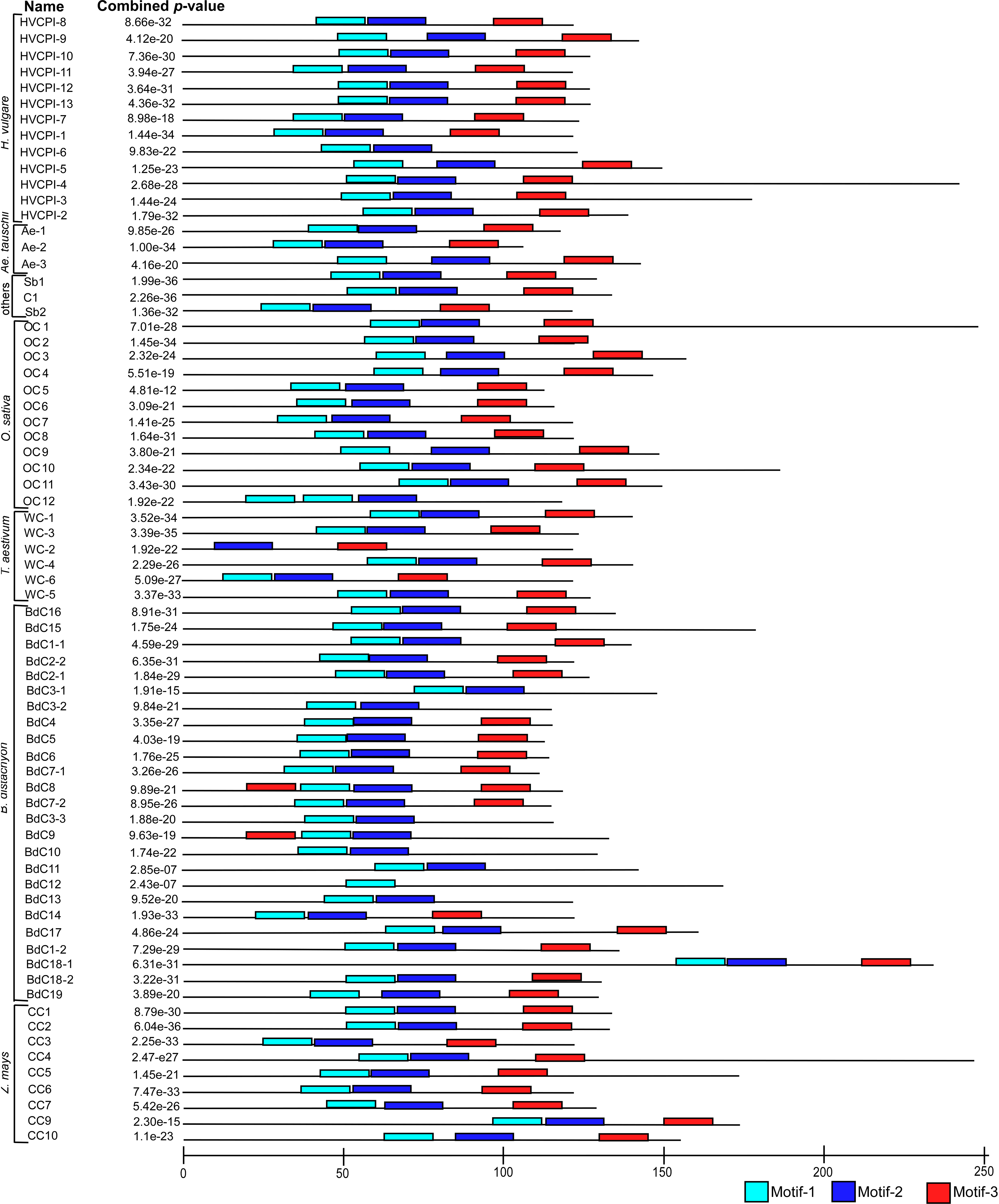

Supplement: Figure S1 — Motifs in cystatin proteins from different plant species identified by MEME analysis. Different colored boxes indicate different motifs and their positions in each cystatin sequence. [file Image1.TIF]

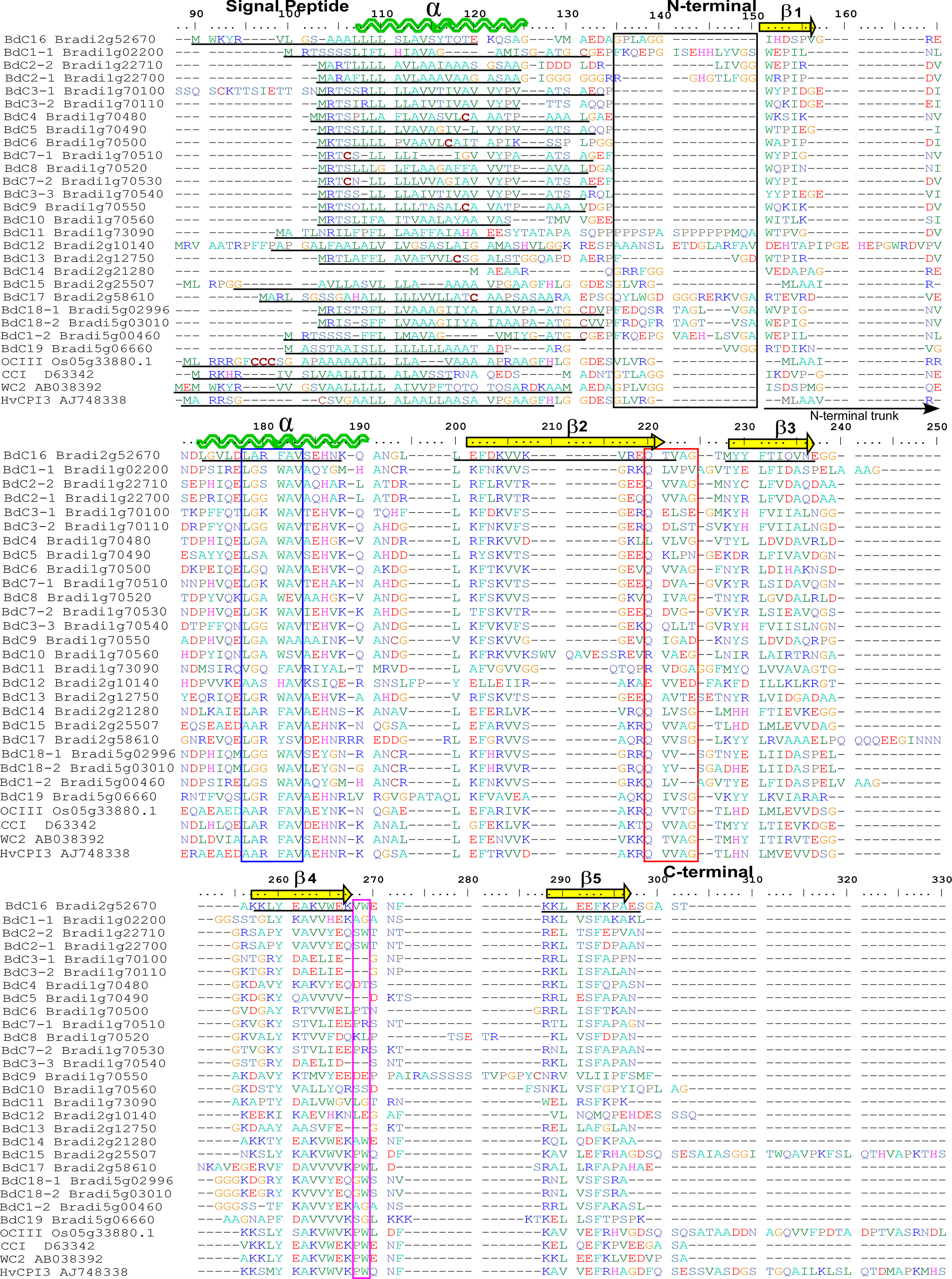

Supplement: Figure S2 — Amino acid clustalW multiple alignment of the 25 members of Brachypodium distachyon cystatins (BdC) along with barely (HvCPI-3), wheat (WC1), rice (OC10) and sorghum (CC1). The locations of the secondary structures (α-helix and β-sheets) are included. Signal peptides are marked by blue shades. The conserved signature sequences of the phy-cys are highlighted by enclosing in colored rectangles (Black, N-terminal G; Blue, LARFAV; Red, QXVXG; Pink, P/AW). Signal peptides are underlined. Few N-terminal (BdC3-1, BdC18-1) and C-terminal (BdC15, OC10, and HvCPI-3) amino acid residues are not shown here. [file Image2.TIF]

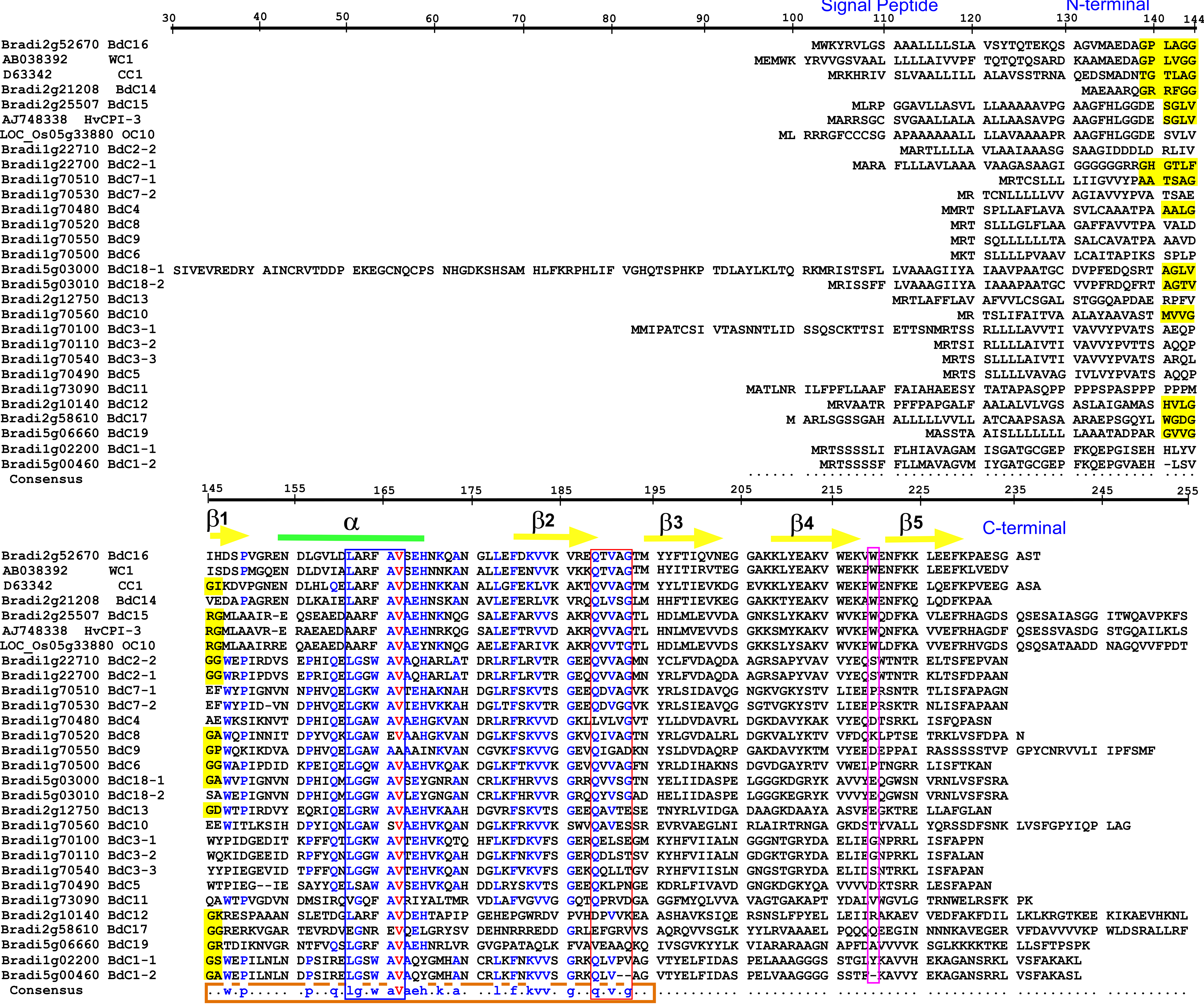

Supplement: Figure S3 — Hierarchical clustering of cystatins from Brachypodium along with related species. The conserved signature motifs are indicated by colored rectangles (Blue, LARFAV; Red, QXVXG; Pink, P/AW). The yellow shaded residues are claimed to be the putative N-terminal “G” residues. The predicted consensus sequences are represented by an orange color box. [file Image3.TIF]

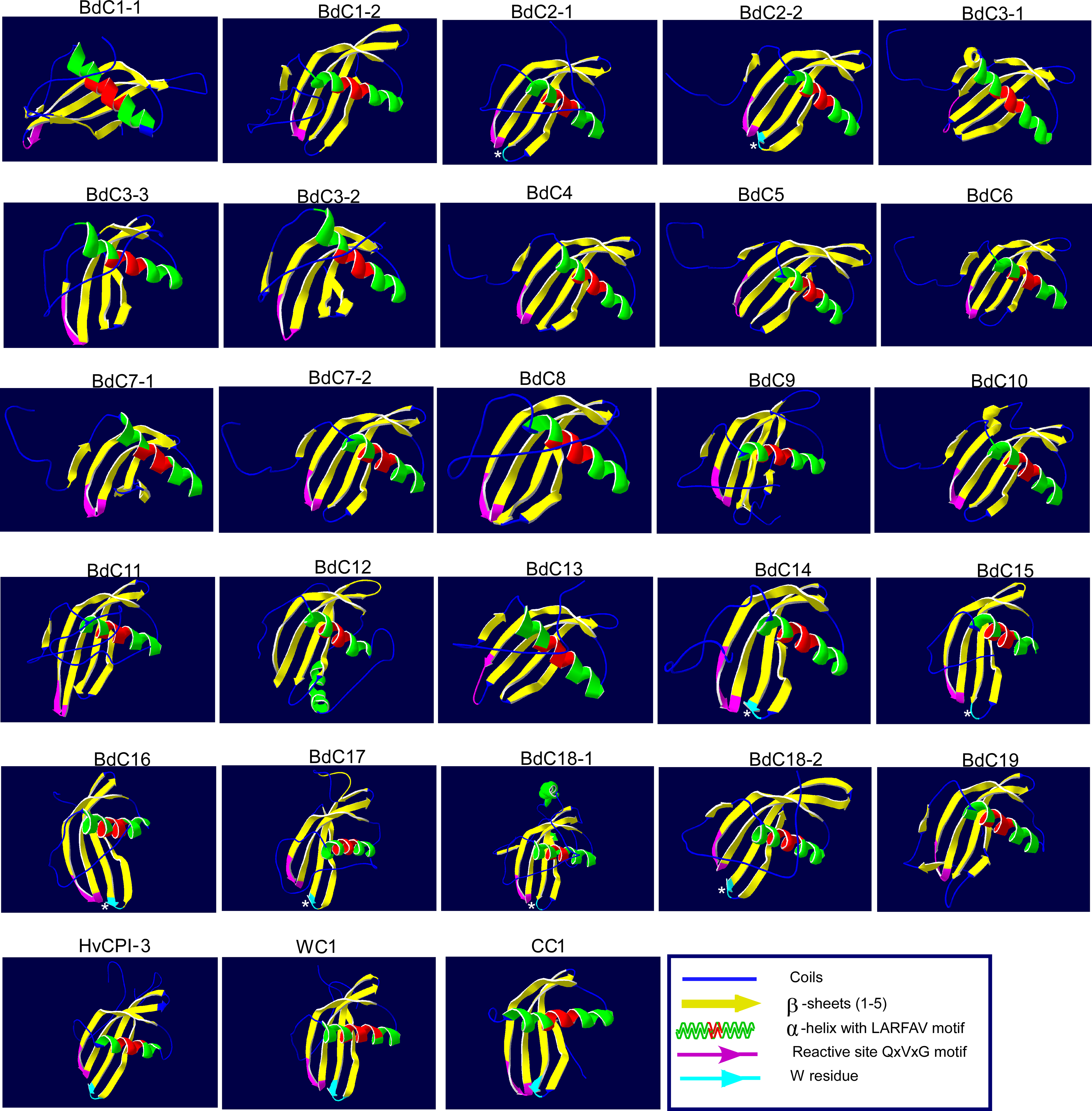

Supplement: Figure S4 — Three dimensional structures of B. distachyon BdC proteins. The tertiary structure was predicted by the Phyre2 server and structure composition indicated similarity with the structure of barely (HvCPI-3), wheat (WC1), and sorghum (CC1) as indicated. The secondary structure is shown with α helixes in green, β sheets in yellow, and loops in blue. [file Image4.TIF]
