# Supplementary material for: Molecular Characterization and Expression Profiling of Brachypodium distachyon L. Cystatin Genes Reveal High Evolutionary Conservation and Functional Divergence in Response to Abiotic Stress
Source: Front Plant Sci. 2017 May 9;8:743. doi: 10.3389/fpls.2017.00743 (PMC5423411; doi:10.3389/fpls.2017.00743)
Supplement: Table S2 — List of Primers designed and used for qRT-PCR analysis of mRNA expression of cystatin genes from B. distachyon (BdC). [file Table2.DOCX]

**Supplementary Table S2** List of Primers designed and used for qRT-PCR analysis of mRNA expression studies of various *cystatin* genes in *B. distachyon* (BdC)

| Gene Name | Melt (Tm °C) | Amplicon size  (bp) | Primer name and sequence  (5’-3’) | Primer binding locations to discriminate between  *BdC* genes |
| --- | --- | --- | --- | --- |
| *BdC1-1* | 60 | 155 | C1F: CTTGATCTTCCTCCACATCG  C1R: GCCCACGTAGAGATGATGCT | 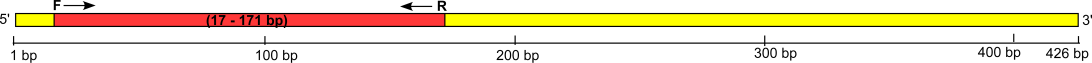 |
| *BdC2-1* | 60 | 181 | C2F: GGACACGGGACGTTGTTC  C2R: CGAAGAGGCGGTAGTTCATC | 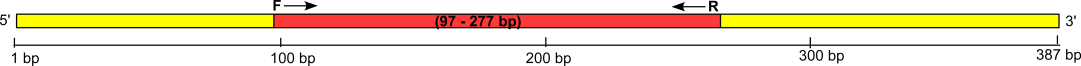 |
| *BdC3-1* | 58 | 142 | C3aF: ATCGACGGAGAGGACATCAC  C3aR: TCATGCCTTCCGATAACTCC | 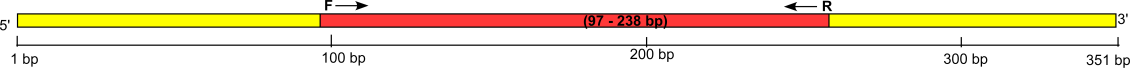 |
| *BdC3-2* | 58 | 200 | C3bF: CGGAGAGGAAATCGACAGAC  C3bR: ACTCCGCATCATACCTACCG | 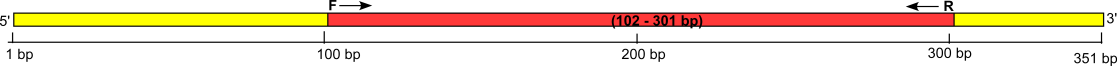 |
| *BdC3-3* | 60 | 186 | C3cF: ACACGCCATTCTTCCAGAAC  C3cR: ACTCCGCATCATACCTACCG | 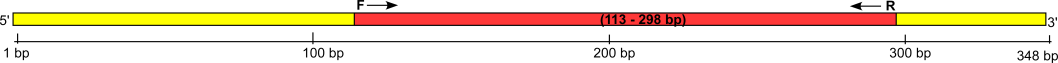 |
| *BdC4* | 60 | 156 | C4F: CAGGCTGAGGTTCAGGAAAG  C4R: AACGAGATGAGCTTGCGACT | 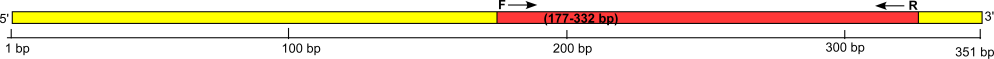 |
| *BdC7-1* | 60 | 159 | C7aF: CATCATCGGCGTGGTTTAC  C7aR: CTGGTCACCTTGCTGAACCT | 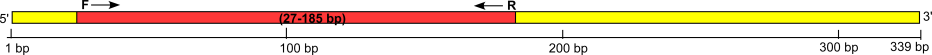 |
| *BdC10* | 60 | 184 | C10F: AGCCTCATATTCGCCATCAC  C10R: CCACCTTCCTGAACTTGAGC | 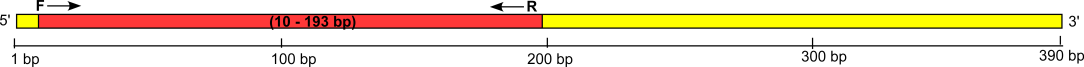 |
| *BdC12* | 60 | 170 | C12F: GAGGTCGAATTCCCTTTTCC  C12R: TCAGCACAAAAGCTCCTTCA | 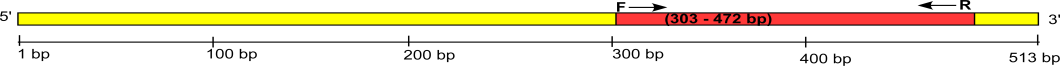 |
| *BdC14* | 60 | 104 | C14F: TACTCCGTGGACGAGCACA  C14R: TTGTTGATTCCCTCCTCCTG | 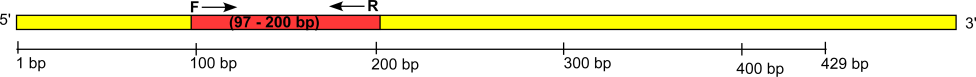 |
| *BdC15* | 60 | 182 | C15F: CTTGCATGACCTGATGTTGG  C15R: AGAACTTGGGAACAGCTTGC | 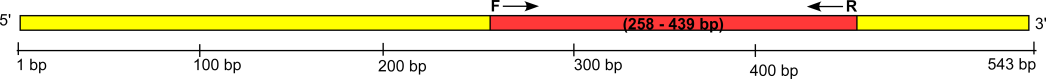 |
| *BdC16* | 60 | 186 | C16F: CTCGCCGTATCGTACACTCA  C16R: ATCGAACTCCAGCAGACCAT | 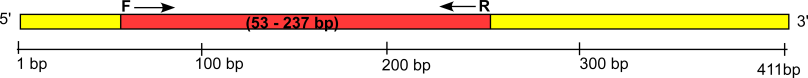 |
| *BdC1-2* | 60 | 146 | C1bF:CAGCTTCTTCCTCCTCATGG  C1bR:GAGAGATGCTCCGCGACTC | 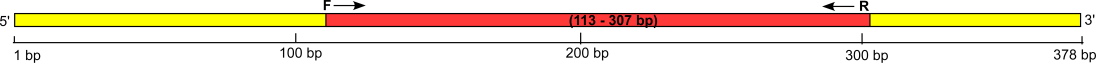 |
| *BdC9* | 60 | 230 | C9F: GCTCCTCCTCCTCCTCCTTA  C9R: GCGTCAACATCAAGCGAGTA | 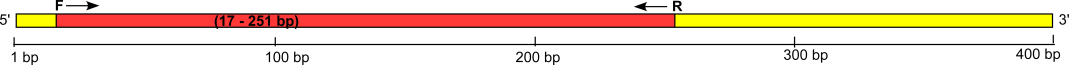 |
| *BdC18-1* | 60 | 157 | C18aF: AGTTCCACAGGGTGGTGAG  C18aR: AAACGAGGTTGCGAACATT | 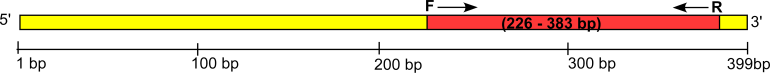 |
| *BdC19* | 60 | 231 | C19F: CAGGAACACATTCGTGCAGT  C19R: CTTCTTCTTGAGCCCCGACT | 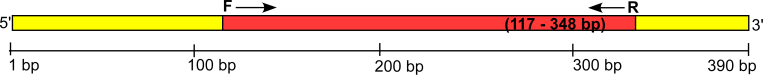 |
| *Ubiquitin4* | 60 | 104 | Ubi1F: TGACACCATCGACAACGTGA  Ubi1R: GAGGGTGGACTCCTTCTGGA | 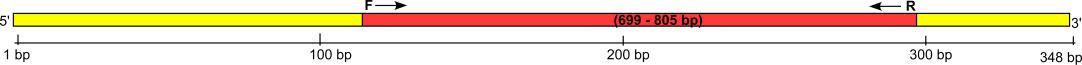 |
