# Supplementary material for: Molecular Characterization and Expression Profiling of Brachypodium distachyon L. Cystatin Genes Reveal High Evolutionary Conservation and Functional Divergence in Response to Abiotic Stress
Source: Front Plant Sci. 2017 May 9;8:743. doi: 10.3389/fpls.2017.00743 (PMC5423411; doi:10.3389/fpls.2017.00743)
Supplement: Table S4 — Absolute quantification of the mRNA expression level of duplicated BdC genes. Quantification was according to the reference gene (Ubiquitin) in the quantitative real-time polymerase chain reaction (qRT-PCR) analysis. The values listed are indicated as cDNA copies/μg of reverse-transcribed total RNA, and the data are shown as the mean ± standard deviation. [file Table4.DOCX]

| **Genes** | **Number of cDNA copies** | | |
| --- | --- | --- | --- |
|  | **Leaf** | **Root** | **Seed** |
| *Ubiquitin* | 1.23×10^8^±1.70×10^6^ | 9.51×10^7^±5.32×10^6^ | 3.97×10^4^±9.40×10^3^ |
| *BdC1-1* | 6.18×10^2^±3.08×10^2^ | 2.88×10^1^±1.37×10^1^ | 2.45×10^1^±1.31×10^1^ |
| *BdC1-2* | 4.27×10^2^±3.43×10^2^ | 3.28×10^1^±2.62×10^1^ | 7.50×10^0^±3.74×10^0^ |
| *BdC3-1* | 1.58×10^1^±6.45×10^0^ | 2.68×10^1^±1.31×10^1^ | 9.20×10^2^±4.29×10^2^ |
| *BdC3-2* | 1.07×10^2^±5.72×10^1^ | 8.17×10^0^±2.58×10^0^ | 2.73×10^1^±1.41×10^1^ |
| *BdC3-3* | 2.20×10^6^±1.20×10^6^ | 3.43×10^5^±1.92×10^5^ | 1.18×10^4^±8.79×10^3^ |

**Supplementary Table S4** Absolute quantification of the mRNA expression level of duplicated *BdC* genes. Quantification was according to the reference gene (*Ubiquitin*) in the quantitative real-time polymerase chain reaction (qRT-PCR) analysis. The values listed are indicated as cDNA copies/μg of reverse-transcribed total RNA, and the data are shown as the mean ± standard deviation.
